# Profiling of the ADP‐Ribosylome in Living Cells

**DOI:** 10.1002/anie.202200977

**Published:** 2022-03-09

**Authors:** Maike Lehner, Sonja Rieth, Eva Höllmüller, Daniel Spliesgar, Bastian Mertes, Florian Stengel, Andreas Marx

**Affiliations:** ^1^ Departments of Chemistry and Biology Konstanz Research School Chemical Biology Universitätsstraße 10 78457 Konstanz Germany

**Keywords:** ADP-Ribosylation, Modified Nucleotides, NAD^+^, Post-Translational Modification, Proteomics

## Abstract

Post‐translational modification (PTM) with ADP‐ribose and poly(ADP‐ribose) using nicotinamide adenine dinucleotide (NAD^+^) as substrate is involved in the regulation of numerous cellular pathways in eukaryotes, notably the response to DNA damage caused by cellular stress. Nevertheless, due to intrinsic properties of NAD^+^ e.g., high polarity and associated poor cell passage, these PTMs are difficult to characterize in cells. Here, two new NAD^+^ derivatives are presented, which carry either a fluorophore or an affinity tag and, in combination with developed methods for mild cell delivery, allow studies in living human cells. We show that this approach allows not only the imaging of ADP‐ribosylation in living cells but also the proteome‐wide analysis of cellular adaptation by protein ADP‐ribosylation as a consequence of environmental changes such as H_2_O_2_‐induced oxidative stress or the effect of the approved anti‐cancer drug olaparib. Our results therefore pave the way for further functional and clinical studies of the ADP‐ribosylated proteome in living cells in health and disease.

## Graphical abstract

For further studies on protein ADP‐ribosylation, two new NAD^+^ derivatives are introduced, which carry either a fluorophore or an affinity tag. In combination with developed methods for their mild cell delivery, studies of ADP‐ribosylation in living human cells were conducted. This approach allows not only the imaging of ADP‐ribosylation in living cells but also the proteome‐wide analysis of cellular adaptation by protein ADP‐ribosylation as a consequence of environmental changes.

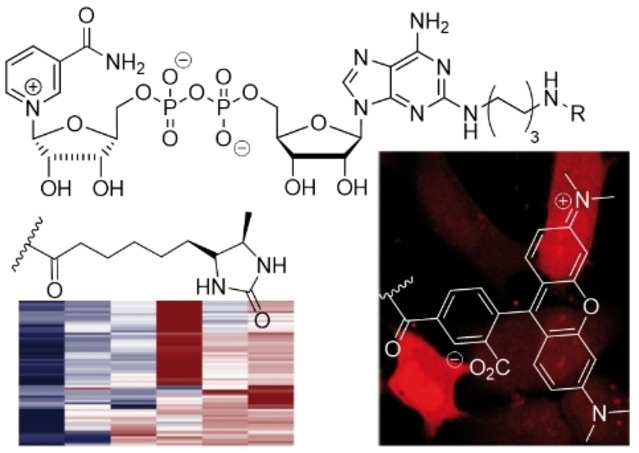

## Introduction

ADP‐ribosylation is a reversible and highly dynamic post‐translational modification (PTM) that was first discovered in nuclear extracts^[1]^ and is known to play important roles in many cellular pathways ranging from bacteria to eukaryotes.^[2]^ Poly‐ADP‐ribose (PAR) is composed of several linear and/or branched ADP‐ribose (ADPr) units which are attached to a substrate protein on diverse amino acid side chains (Glu, Asp, Arg, Cys, Lys, Ser, Tyr).^[3]^ The attachment is catalyzed by the enzyme family of ADP‐ribose transferases (ARTs), using nicotinamide adenine dinucleotide (NAD^+^) as substrate. Thereby, one or several ADP‐ribose moieties are transferred to a substrate protein to obtain mono‐ADP‐ribosylated (MARylated: catalyzed by ARTD3, 4, 7–12, 14–17) or poly‐ADP‐ribosylated (PARylated; ARTD1, 2, 5, 6) targets.[^2c^, ^4^] This protein modification is dynamic and reversed by poly(ADP‐ribose) glycohydrolases (PARG) or (ADP‐ribose) hydrolases (ARH).[^2^, ^5^] Due to a dramatic change of electrostatic property of the acceptor protein by PAR attachment, target proteins are affected in their structure, function and interaction network.[^2^, ^6^] This leads to the regulation of various biological processes by ADP‐ribosylation, for example translational control, cell signaling, cell division and DNA repair.[^3a^, ^7^]

Depending on their conserved structural features, there are two major subclasses of ARTs: The ARTCs (cholera toxin like) and the ARTDs (diphtheria toxin‐like; also known as poly(ADP‐ribose) polymerase, PARPs).^[8]^ ARTD1 (also known as PARP1), which is the most prominent member of the ARTD family, is localized in the nucleus.^[9]^ In case of DNA strand breaks, ARTD1 is activated and starts to auto‐poly‐ADP‐ribosylate itself as well as PARylation of a large number of proteins. Thereby the recruitment of several enzymes is ensued for the initiation of the DNA damage repair mechanism.[^9^, ^10^] Due to this important role in genomic maintenance, PARylation has emerged as an important target for the development of therapeutics against cancer.^[11]^ Olaparib is a nanomolar inhibitor of ARTD1 and ARTD2 (PARP2) that is approved by the FDA (U.S. Food and Drug Administration) and the EMA (European Medicines Agency) for the treatment of patients with ovarian, fallopian tube or primary peritoneal cancer.^[12]^ In homologous recombination (HR)‐deficient cancer cells olaparib acts through synthetic lethality by enhancement of genomic instability caused by oxidative and replication stress though inhibition of compensatory DNA repair pathways.[^12a^, ^13^] Even though ADP‐ribosylation is implicated in the regulation of a vast variety of important biological processes, our knowledge of PAR functions remains sparse, which is mainly caused due to the lack of appropriate tools for a biochemical analysis of this dynamic and complex PTM. Mass spectrometry‐based proteomics has been shown to be a promising tool in the elucidation of the ADP‐ribosylated proteome.^[14]^ Various approaches using antibody^[15]^‐, macro domain^[16]^‐ or boronate^[4b]^‐based affinity chromatography were successfully employed to obtain proteome wide‐data for ADP‐ribosylation. However, these approaches suffer from one or several of the following aspects that make research on PAR so challenging: the length dependence of the PAR‐binding proteins, co‐enrichment of non‐covalent PAR‐binding proteins, inherent dynamics of ADP‐ribosylation itself that require additional measures to counterbalance the action of the PAR‐degrading enzyme PARG.^[16a]^ Furthermore, insights into the background signal for PARylation independent of external cues are hard to derive from these studies, since cell rupture that is required to prepare the protein samples for subsequent analysis, is a stressor that affects ADP‐ribosylation, thus confounding the obtained experimental results.

To alleviate these problems and to study ADP‐ribosylation, multiple approaches based on bio‐orthogonal chemistry were also developed. Several NAD^+^ analogs were developed and employed but, due to the lack of cell permeability of these NAD^+^ analogs, only in vitro experiments e.g., in cell extracts were so far feasible.^[17]^ In addition, many approaches that employ NAD^+^‐analogs are based on Copper‐catalyzed alkyne‐azide “Click” chemistry (CuAAC),^[18]^ whose reaction kinetics (second‐order rate constants of 10–200 M^−1^ s^−1^) are concentration dependent^[19]^ and thus will fail to trap less abundant proteins. Furthermore, the modifications are often introduced at positions that significantly interfere with processing of ARTDs.^[20]^ These issues might have been causative for the fact that some proteins that are known targets for PARylation such as ARTD2^[21]^ have not been identified in some approaches.

**Figure 1:**
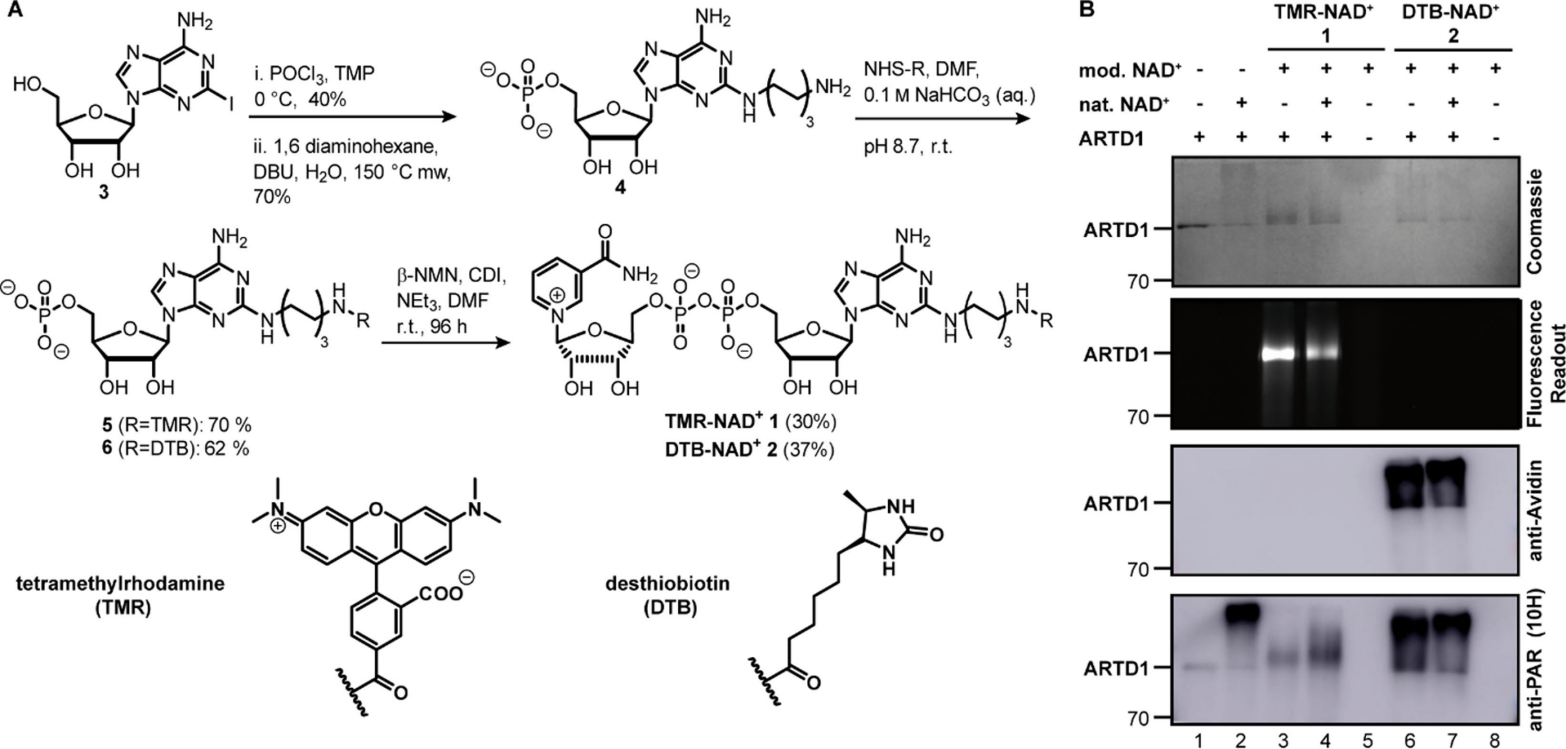
A) Synthesis of the new NAD^+^ analogs **TMR‐NAD^+^ 1** and **DTB‐NAD^+^ 2** starting from 2‐iodoadenosine (**3**) as precursor. TMP=trimethyl phosphate, DBU=1,8‐diazabicyclo[5.4.0]undec‐7‐ene, *β*‐NMN=*β*‐nicotin‐amide mononucleotide, CDI=*N*,*N*‐carbonyldiimidazole. B) SDS PAGE and western blot analysis of auto‐ADP‐ribosylation with ARTD1 using **TMR‐NAD^+^ 1** or **DTB‐NAD^+^ 2** thereby ARTD1 serves as its own acceptor. The total concentration of NAD^+^ in each reaction was 1 mm. The NAD^+^ derivatives were also tested in 1 : 1 ratio with natural NAD^+^ (lane 4 and 7). Controls were performed using either natural NAD^+^ (lane 2) or no enzyme (lane 5 and 8).

Recently, an approach to identify targets of ADP‐ribosylation in cells was reported in which a cell‐permeable modified adenosine nucleoside analog was utilized.^[22]^ The modified adenosine was metabolized also to NAD^+^ and used by the cellular machinery for ADP‐ribosylation. However, modified ATP is also formed by metabolic processing with these analogs which complicates analysis due to simultaneous enrichment of ATP‐derived PTMs like AMPylated proteins.^[23]^ We have recently reported that even bulky modifications such as tags for copper‐free bio‐orthogonal chemistry^[20a]^ or dyes[^20b^, ^20c^] at position *C*2 on the adenine core of the NAD^+^ are well accepted by ARTDs while other positions are less well tolerated. However, this approach suffered from a low cell viability after transfection of the charged NAD^+^ analog using the carrier peptide Pep‐1. Here we report on novel NAD^+^ analogs (Figure 1A) that can be delivered into cells without compromising their vitality allowing the investigation of conditional ADP‐ribosylation in living human cell lines. The new *C*2 modified NAD^+^ bearing a fluorophore allows for the visualization of ADP‐ribosylation in living cells while concomitantly the desthiobiotin‐modified analog enables proteome‐wide enrichment and subsequent profiling of ADP‐ribosylated proteins by mass spectrometry‐based proteomics. We employed this approach to study the effects of endogenous cues such as oxidative stress or the approved drug olaparib on the ADP‐ribosylated proteome of living HeLa cells. We found that some proteins are already ADP‐ribosylated in the absence of any cellular stress (i.e. basal conditions). Upon H_2_O_2_‐induced oxidative stress, the number of ADP‐ribosylated proteins increased significantly and mainly nuclear proteins involved in DNA repair and replication were enriched, confirming ADP‐ribosylation as a stress signal. Interestingly, when H_2_O_2_‐treated cells were incubated with olaparib, a known inhibitor of ARTDs 1 and 2, the nature of the enriched proteins changed dramatically, leading to the enrichment of proteins that are involved in RNA‐related processes. This highlights the interrelation of ADP‐ribosylation with transcription and follow‐up processes like RNA processing and might also point towards side effects of the drug.

Taking together, our new methodology for the cellular uptake of novel modified NAD^+^ derivatives enables imaging of PAR in living cells. Furthermore, it allows the identification of ADP‐ribosylated target proteins under different physiological conditions.

## Results

### Synthesis of Modified NAD^+^ Analog 1 and Its Acceptance by ARTD1

NAD^+^ analogs that bear bulky substitutions at the *C*2‐position of the adenine have successfully been applied in ADP‐ribosylation reaction by ARTDs (e.g., ARTD1/2) previously.^[20]^ Here, we report two new *C*2‐modified NAD^+^ analogs: One bearing the dye tetramethylrhodamine (**TMR‐NAD^+^ 1**) and one bearing the affinity tag desthiobiotin (**DTB‐NAD^+^ 2**, Figure 1A). Both analogs are synthesized more efficiently than the earlier described molecules. The synthesis starts with commercially available 2‐iodoadenosine **3** (Figure 1A). Phosphorylation of the primary 5′‐OH and subsequent substitution of the iodide with 1,6 diaminohexane resulted in **4**. The dye and the affinity labels were introduced by NHS chemistry to afford the intermediates **5** and **6**, respectively. Condensation with *β*‐nicotinamide mononucleotide (*β*‐NMN)^[24]^ afforded the target compounds **TMR‐NAD^+^ 1** and **DTB‐NAD^+^ 2**, respectively.

With both compounds in hand, we first tested **TMR‐NAD^+^ 1** in an ARTD1 auto‐modification assay for its substrate acceptance.^[24]^ Briefly, **TMR‐NAD^+^ 1** was incubated with recombinant ARTD1 and short dsDNA as its activator, followed by SDS‐PAGE and western blot analysis (Figure 1B and Supporting Information Figures 1, 2). The obtained data show that the analog is a substrate for ARTD1 and the protein modified with a characteristic fluorescent signals. In comparison to the natural NAD^+^, the processing of **TMR‐NAD^+^ 1** results either in shorter PAR chains or mono‐ADP‐ribosylation at several sites. This suggests that the modification might affect the chain elongation. However, by applying a 1 : 1 mixture of modified NAD^+^ with natural NAD^+^, we detected a heterogeneous product formation (Figure 1B, lane 4). This might be caused either by the formation of longer PAR chains that contain the fluorescent building block or by mono‐ADP‐ribosylation at several sites.

### Cellular Internalization of TMR‐NAD^+^ 1 and Metabolic Labeling of ADP‐Ribosylation in Living Cells

**Figure 2:**
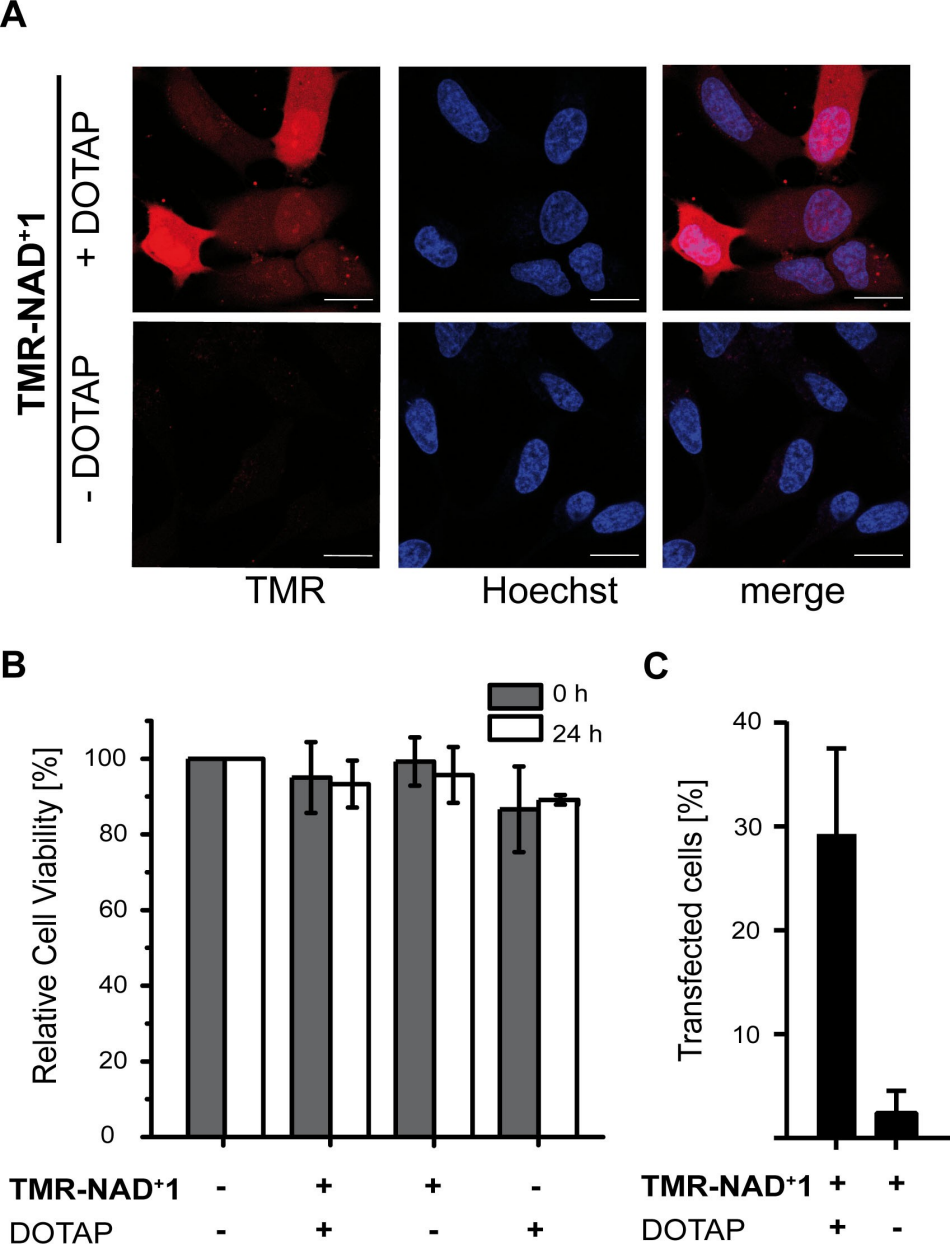
Validation of the cellular internalization by **TMR‐NAD^+^ 1**. A) HeLa cells were incubated with **TMR‐NAD^+^ 1**, DOTAP transfection mixture and analyzed by confocal microscopy. Controls were performed without the transfection reagent DOTAP. (Scale bar: 20 μm). B) Plot showing the cell viability of HeLa cells in the presence of the developed transfection method using **TMR‐NAD^+^ 1**. HeLa cells (5000 cells per well) were seeded in a 96‐well plate and the transfection method was used to transfect **TMR‐NAD^+^ 1**. The cell viabilities in the presence of mod. NAD^+^ are reported relative to cell viability of HeLa cells without treatment as a control. C) Transfection efficiency of **TMR‐NAD^+^ 1** determined by flow cytometry compared to cells without the transfection reagent DOTAP. Cells treated with the developed transfection method showed an over 10‐fold increased uptake of **TMR‐NAD^+^ 1**.

**Figure 3:**
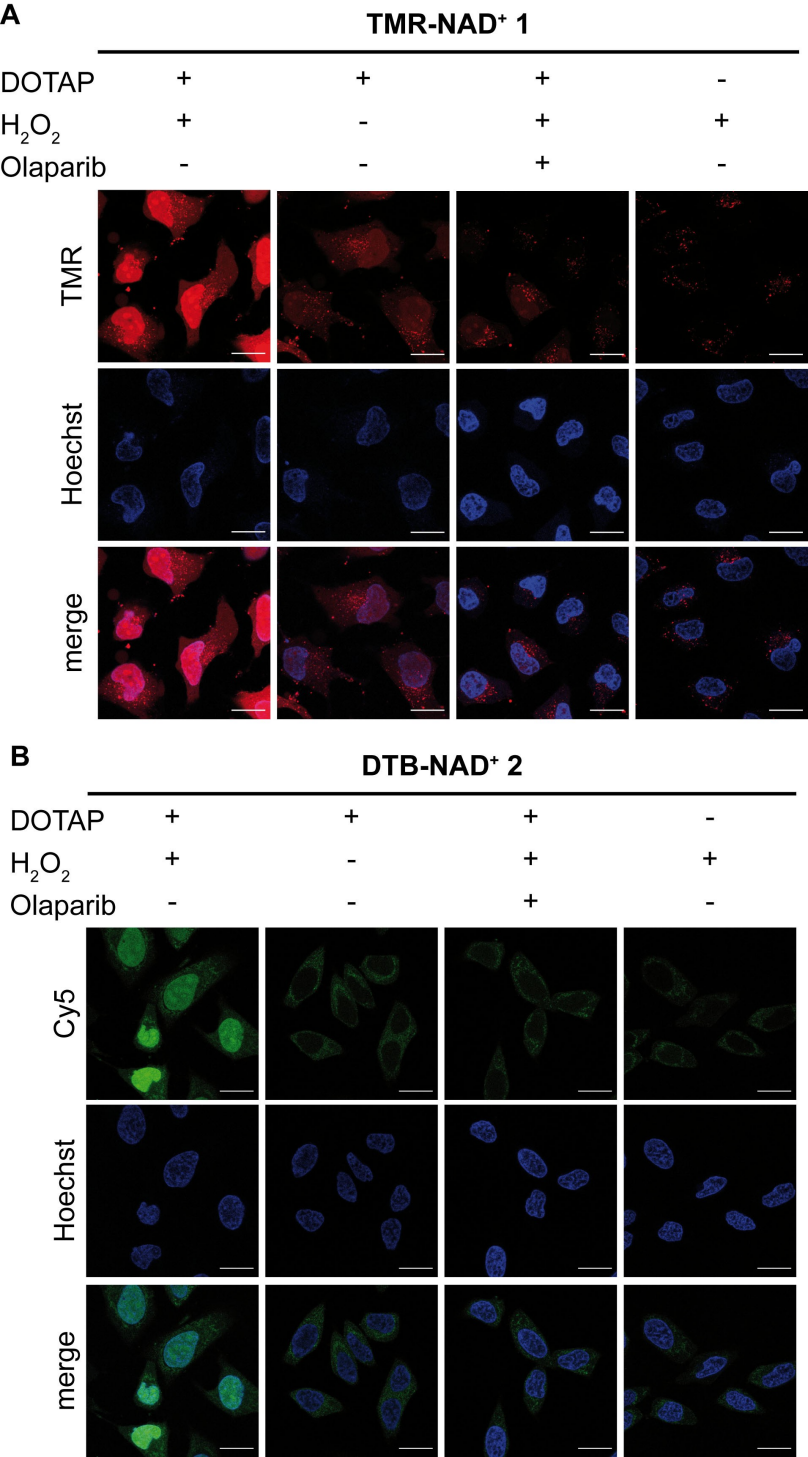
Visualization of endogenous PAR formation using **TMR‐NAD^+^ 1** and **DTB‐NAD^+^ 2**. HeLa cells were incubated with A) **TMR‐NAD^+^ 1**, DOTAP or B) **DTB‐NAD^+^ 2**, DOTAP transfection mixture, stressed with H_2_O_2_ as indicated and fixed. For the **DTB‐NAD^+^ 2**, the cells were transiently permeabilized and stained with Strep‐Cy5. Controls were performed without H_2_O_2_ treatment, with inhibitor Olaparib and without the transfection reagent DOTAP. Samples were analyzed by CLSM. Scale bar: 20 μm.

After positive evaluation of the acceptance of **TMR‐NAD^+^ 1** in in vitro assays, we examined its cellular applications. First, we aimed at identifying suitable means for the cellular uptake of **TMR‐NAD^+^ 1** and tested different transfection reagents. After screening several transfection reagents (for details see Supporting Information) we identified DOTAP (1,2‐dioleoyl‐3‐trimethyl‐ammonium propane) being best suited for our purpose. For HeLa cells we found that incubation with the transfection mixture (50 μm **TMR‐NAD^+^ 1**, 15 μm DOTAP) for 1 hour led to the best result regarding transfection efficiency. Subsequently, imaging by fluorescence microscopy was conducted (Figure 2A, Supporting Information Figure 3). Transfection of **TMR‐NAD^+^ 1** with DOTAP showed an evenly distributed fluorescent signal in HeLa cells in comparison to the incubation without the transfection reagent (Figure 2A) combined with barely any toxicity 24 h after transfection (Figure 2B). Furthermore, the transfection efficiency was determined with flow cytometry showing an over 10‐fold increased uptake of **TMR‐NAD^+^ 1** in the presence of DOTAP in comparison to the absence of DOTAP usage (Figure 2C, Supporting Information Figure 4). To evaluate the propensity of metabolic labeling of intracellular ADP‐ribosylation by **TMR‐NAD^+^ 1**, HeLa cells were transfected with the analog and subsequently treated with H_2_O_2_ ^[25]^ to induce oxidative stress and stimulation of ADP‐ribosylation. The cells were fixed and ADP‐ribosylation in the nucleus was observed visualized by confocal fluorescence microscopy (Figure 3A). As controls, cells were either incubated only with **TMR‐NAD^+^ 1** in absence of DOTAP but with H_2_O_2_ or incubated with the ARTD inhibitor olaparib^[12b]^ prior to H_2_O_2_ treatment. Conclusively, these results demonstrate the efficient and highly viable cellular uptake of the **TMR‐NAD^+^ 1** using DOTAP as well as the metabolic labeling of ADP‐ribosylation by the usage of **TMR‐NAD^+^ 1** by endogenous ARTDs in living cells.

It should be noted that we obtain an analog‐dependent signal even though we add lower concentrations of NAD^+^ analogs (50 μM) to cells than occur naturally in cells (0.2–0.5 mM^[7c]^). This shows that the NAD+ analogs can be introduced into the cell at physiologically relevant concentrations.

### Studies of NAD^+^ Analog 2 Bearing an Affinity Tag

Next, we investigated **DTB‐NAD^+^ 2**. Due to the limitations of CuAAC and the scope of acceptance of ARTDs of *C2*‐modified adenosines in NAD^+^ as discussed above, we used desthiobiotin (DTB) as affinity tag since enrichment can be conducted by streptavidin‐beads (desthiobiotin‐streptavidin *K*_D_ is approx. 10^−13^ M) and under mild elution conditions with biotin (biotin‐streptavidin *K*_D_ is approx. 10^−15^ M).^[26]^ Substrate acceptance of the **DTB‐NAD^+^ 2** by ARTD1 in vitro was investigated as described for **TMR‐NAD^+^ 1** above. The resulting western blot analysis using avidin‐horse radish peroxidase (HRP) conjugate or an anti‐PAR (10H) antibody and the corresponding Coomassie Blue stain are shown in Figure 1B. The intensity of the band derived from ARTD1 in the Coomassie Blue stained gel was decreased in comparison to the control in lane 1. Heterogeneous polymer formation was observed in the Coomassie Blue stained gel as well as in the western blot analysis with avidin‐HRP conjugate, suggesting that ARTD1 was PARylated. In comparison with the unmodified NAD^+^, ADP‐ribosylation using **DTB‐NAD^+^ 2** results in shorter PAR chains, but in comparison to the **TMR‐NAD^+^ 1** longer PAR chains were formed. This suggests that the modification in **DTB‐NAD^+^ 2** has less impact on the PAR chain formation than **TMR‐NAD^+^ 1**. Moreover, the 1 : 1 mixture of modified **DTB‐NAD^+^ 2** with unmodified NAD^+^ (Figure 1B, lane 7) exhibited polymer formation in the western blot analysis with the avidin‐HRP antibody, suggesting that **DTB‐NAD^+^ 2** can also be used as substrate for protein ADP‐ribosylation.

**Figure 4:**
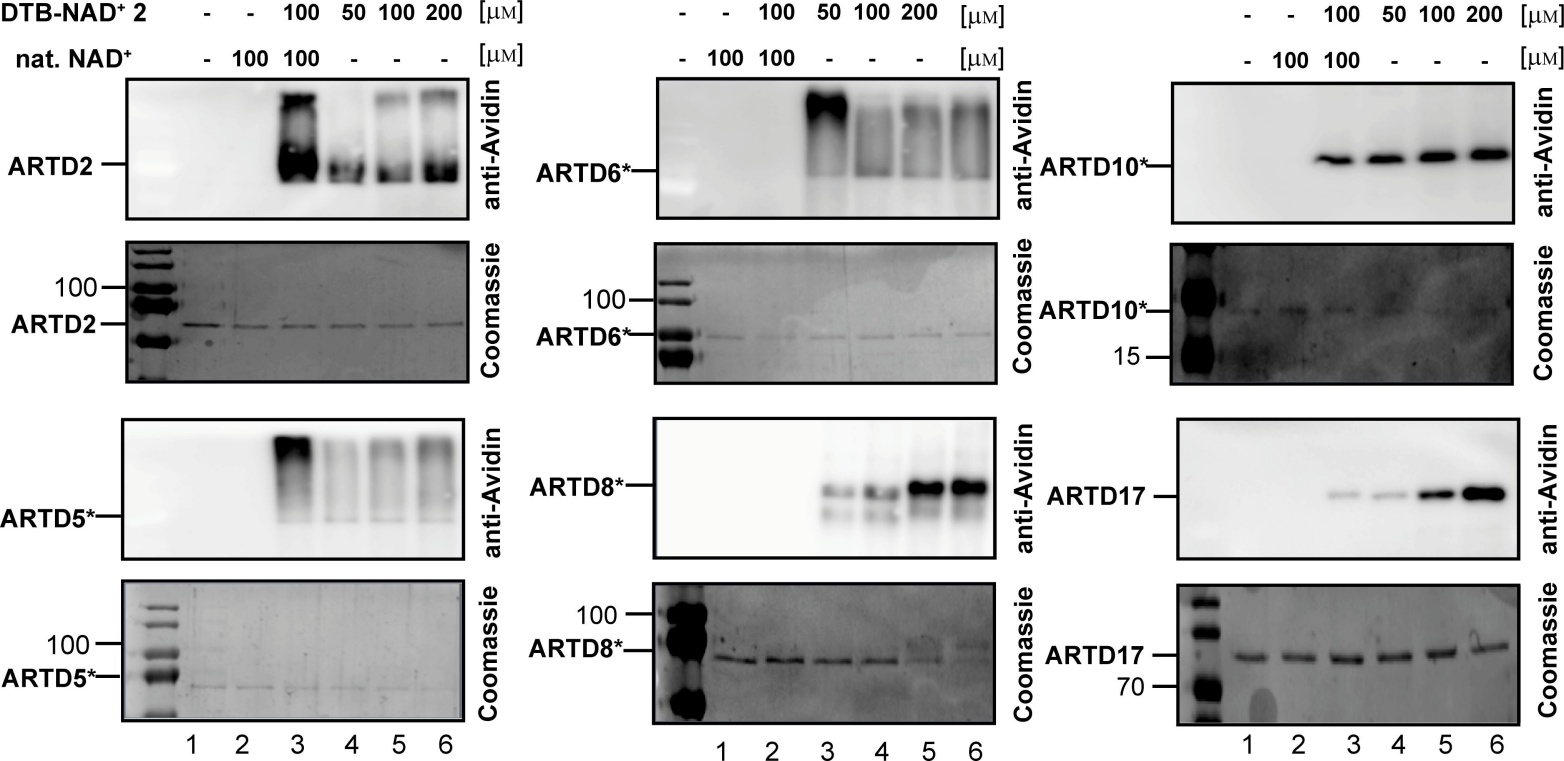
Acceptance of **DTB‐NAD^+^2** by different ARTD‐members investigated via an auto‐ADP‐ribosylation assay. SDS‐Page and western blot analysis of the respective member are shown. Reactions were performed using only **DTB‐NAD^+^2** (lane 4, 5, 6) and a 1 : 1 ration of natural NAD^+^ and **NAD‐DTB‐NAD^+^2**. Controls were performed, without NAD^+^ and with natural. NAD^+^ (lane 1 and 2). The applied concentrations are indicated above the respective lanes. *No full‐length protein, mainly catalytic domain.

**Figure 5:**
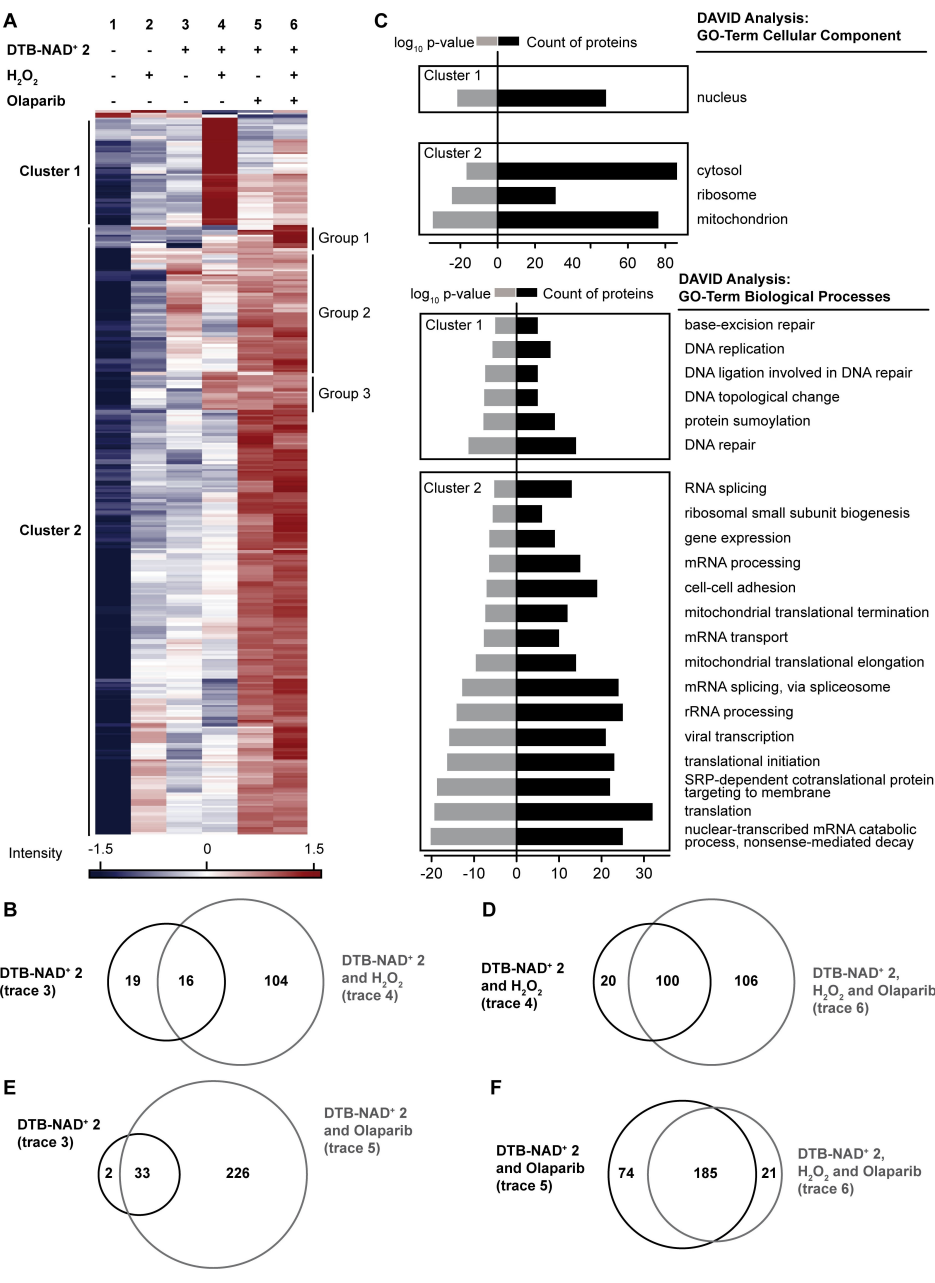
Statistical analysis of ADP‐ribosylated proteins using **DTB‐NAD^+^ 2**. A) Heatmap of endogenous ADP‐ribosylated proteins in HeLa cells. 310 significantly enriched targets were identified by LC‐MS/MS analysis and label free quantification (FDR: 0.002; S_0_=2). B) Venn diagram showing the overlap between ADP‐ribosylated targets from untreated (trace 3) and cells treated with H_2_O_2_ (trace 4). C) GO‐term analysis: proteins significantly enriched in ANOVA analysis were classified according to cellular components and biological processes using DAVID (complete dataset see Supporting Information). D–F) Venn diagrams showing the overlap between ADP‐ribosylated targets found in the indicated experiments.

After showing the acceptance of the **DTB‐NAD^+^ 2** by ARTD1, we continued testing different ARTDs, namely ARTD2, 5, 6, 8, 10 and 17 (Figure 5). **DTB‐NAD^+^ 2** was accepted by all tested ARTDs, also in a 1 : 1 mixture with natural NAD^+^ (Figure 4). This shows that **DTB‐NAD^+^ 2** has a brought applicability and can be applied to investigated further ARTDs.

Subsequently, cell delivery and metabolic labeling of endogenous ADP‐ribosylation was examined. Therefore, the transfection method of **TMR‐NAD^+^ 1** was adapted for the transfection of **DTB‐NAD^+^ 2**. The ADP‐ribosylation catalyzed by endogenous ARTDs was evaluated by transfecting HeLa cells with **DTB‐NAD^+^ 2** using DOTAP. Upon subsequent H_2_O_2_ treatment, fixation, permeabilization and staining with a streptavidin‐Cy5 conjugate, increased nuclear staining was observed by fluorescence microscopy (Figure 3B). Like **TMR‐NAD^+^ 1**, the signal for ADP‐ribosylation was largely abolished by the pre‐incubation with olaparib, both in absence of DOTAP and without H_2_O_2_treatment. Additionally, a low toxicity was confirmed by cell viability tests using the optimized transfection conditions for the **DTB‐NAD^+^ 2** (Supporting Information Figure 3). Overall, these results demonstrate the successful cellular uptake of **DTB‐NAD^+^ 2** and in cell labeling of PAR by endogenous ARTDs using a novel mild transfection method.

### Analysis of the ADP‐Ribosylome in Living Cells in Response to External Cues

Having established the synthesis of **DTB‐NAD^+^ 2** and its endogenous processing to form ADP‐ribosylated proteins we demonstrated that ADP‐ribosylated proteins can be enriched with streptavidin‐agarose beads (Supporting Information Figure 5). Based on these findings we now established a workflow for the proteomic analysis of proteins that are ADP‐ribosylated by usage of **DTB‐NAD^+^ 2** as substrate (Supporting Information Figure 6). In brief, after the cellular internalization of **DTB‐NAD^+^ 2** using the established transfection protocol, HeLa cells were left either untreated, treated with the drug olaparib or with H_2_O_2_ in the absence or presence of olaparib and subsequently lysed to isolate the modified proteins. Enrichment of the ADP‐ribosylated proteins was performed using streptavidin‐agarose beads, followed by several stringent steps (e.g., 1 % SDS and 4 m urea) to wash away non‐DTB tagged proteins. Subsequently, DTB‐modified proteins were eluted by using biotin (Supporting Information Figure 5).

To facilitate protein identification by LC‐MS/MS analysis, the isolated proteins were treated with PARG to degrade the PARylated targets.^[5a]^ The samples were resolved by SDS‐PAGE analysis (Supporting Information Figure 5). After tryptic digest, elution fractions were analyzed by LC‐MS/MS followed by label‐free quantification.^[27]^ All proteomics experiments were carried out in triplicates and each sample was measured twice. Thereby we obtained a total of 2 067 proteins that were reliably identified in at least five of the six measurements. After statistical validation (ANOVA statistics, FDR=0.002, S0=2) 310 significantly enriched proteins were identified (Figure 5A).

In order to evaluate the results obtained by our MS analysis, we confirmed the identification of several ADP‐ribosylated protein targets by western blot analysis (Supporting Information Figure 7). The obtained results were in agreement with our proteomic data. Overall, these results show that the chemical proteomic approach for the identification of covalently labeled ADP‐ribosylated proteins developed in this study is robust and viable for the identification of known and new proteins that get conditionally ADP‐ribosylated.

Next, to access the background ADP‐ribosylation in the absence of any external stressors we performed an experimental set in the absence of both H_2_O_2_ or any drug and identified 35 proteins under basal levels (Figure 5A, trace 3, Figure 5B, Supporting Information Figure 8) that were enriched in comparison to the control reactions (conducted in the absence of any **DTB‐NAD^+^ 2** (Figure 5A, traces 1 and 2)). When H_2_O_2_‐induced oxidative stress was imposed on the cells the number of proteins that were enriched increased to 120 (Figure 5A, compare traces 3 vs. 4). Interestingly, 16 proteins were significantly enriched in both cases but 104 were significantly enriched only when cells were treated with H_2_O_2_ (Figure 5B). When the drug olaparib, an inhibitor of ARTD1 and 2,^[12b]^ was applied to cells that were treated with H_2_O_2_, the enrichment pattern significantly changed and a surprisingly large number of a total of 206 proteins were significantly enriched (Figure 5A, compare traces 4 vs. 6). Interestingly, 100 proteins were enriched under both conditions of H_2_O_2_ treatment in the absence and presence of olaparib (Figure 5D). The enriched proteins were grouped in two clusters as indicated in Figure 5A. Cluster 1 (63 proteins) includes proteins which were ADP‐ribosylated after cells were exposed to H_2_O_2_ oxidative stress. Here, ADP‐ribosylation was strongly repressed by olaparib treatment and consequently, Cluster 2 (244 proteins) contains proteins that showed a high intensity of ADP‐ribosylation after olaparib treatment. We then used DAVID^[28]^ (Database for Annotation, Visualization and Integrated Discovery) to identify gene ontology (GO) terms for both clusters (Figure 5C, Supporting Information Figure 9). GO analysis of the targets of cluster 1 revealed that most of the proteins were located in the nucleus and involved in DNA‐dependent processes like DNA repair and replication. In comparison, the biological processes associated with cluster 2 were mainly located in the cytosol, mitochondria, and at the ribosome and are involved in translation, viral transcription, RNA processing, transport or splicing.

We also investigated the effect of the drug olaparib on ADP‐ribosylation in the absence of any H_2_O_2_‐induced oxidative stress (Figure 5A, trace 5). Much to our surprise, we found that the largest set of proteins (namely 259) were enriched in comparison to the controls in these experiments. 33 of the proteins were also found in the background ADP‐ribosylation (Figure 5A, compare traces 3 vs. 5) when no drug was administrated (Figure 5E). A large intersecting‐set of 185 significantly enriched proteins (mainly cluster 2) was observed between the two experiments conducted in the presence of olaparib. However, 74 significantly enriched proteins were identified exclusively when only olaparib was administered and 21 when the drug and H_2_O_2_ were added (Figure 5F). GO analysis revealed differences between the cohorts and the biological processes they are involved in (Supporting Information Figure 10). The large 185 protein containing subset was enriched in proteins that are involved in RNA related processes like transcription, ribosome biogenesis to mRNA processing and translation. The 21 protein containing subset is enriched in proteins involved in DNA ligation and its topological changes while the 74 protein containing subset in the tricarboxylic acid cycle and amino acid biosynthetic processes.

## Discussion

ADP‐ribosylation is a ubiquitous and complex PTM that is involved in a vast variety of biological processes. However, the lack of sensitive functional tools and methods that in particularly can be applied to living cells hamper the progress of studying its biological roles. Herein, we have demonstrated that NAD^+^ derivatives are taken up by the cell and can be employed to monitor intracellular ADP‐ribosylation and the conditional identification of ADP‐ribosylated targets in living human cells.

First, we developed a protocol for the cellular uptake of the fluorescent **TMR‐NAD^+^ 1**. By incubation of HeLa cells with **TMR‐NAD^+^ 1** in the presence of the transfecting reagent DOTAP an even distribution of fluorescent signal was shown (Figure 2A). This is in stark contrast to the results obtained when DOTAP was excluded which gave rise to stained vesicles that most likely resulted from cellular uptake via endocytosis. Another important characteristic of the developed transfection method is the high cell viability in comparison to the previously applied method for the cellular uptake of a fluorescent labeled NAD^+^ analog (Supporting Information Figure 3).^[20b]^ Notably, DOTAP was also reported to promote the cellular uptake of a nucleotide analog with low cell toxicity.^[29]^ Upon DNA damage induction through H_2_O_2_, *in cell* visualization of metabolic labeling of ADP‐ribosylated proteins by **TMR‐NAD^+^ 1** could be followed (Figure 3A). This demonstrates that the analog is a competitive substrate in presence of natural NAD^+^ and our delivery method is a robust approach for metabolic labeling with a fluorescent reporter. These features as well as the improved synthesis method make **TMR‐NAD^+^ 1** a powerful tool for further applications like real‐time imaging of effectors of PAR formation like protein and drugs.^[20b]^ Encouraged by these findings, we developed a new probe for proteomic profiling of ADP‐ribosylated proteins. Thus, we synthesized a new NAD analog that bears a desthiobiotin affinity tag that allows for the sensitive enrichment of ADP‐ribosylated proteins using streptavidin‐agarose beads and mild elution by outcompeting of the targets with biotin. The **DTB‐NAD^+^ 2** was first tested for its acceptance by ARTD1, 2, 5, 6, 8, 10, 17 in in vitro assays as well as in in cellulo experiments using the established cell delivery method. Also **DTB‐NAD^+^ 2** is a competitive substrate of natural NAD^+^ in a cellular environment. Furthermore, we were able to profile the conditional ADP‐ribosylome as a function of stimulation by various external cues like H_2_O_2_ and olaparib and initially identified 2 067 proteins that were consistently identified in each of the tested conditions. Applying ANOVA statistics resulted in 310 significantly enriched target proteins of ADP‐ribosylation, which were modified under different physiological conditions. Our study show varied overlap to several reports before that were mostly done in cell lysates (Supporting Information Figure 11). Overall, 73 % of the proteins found in this study to be modified were also reported before.

In order to access the basal ADP‐ribosylation, we treated cells with **DTB‐NAD^+^ 2** in the absence of any external stimulus and identified 35 proteins that were significantly enriched (Figure 5). A GO analysis revealed that besides proteins that are involved in protein auto‐ and poly‐ADP ribosylation (including ARTD8 (PARP14), ARTD 10 (PARPs 10), ARTD5 (tankyrase 1), and the poly(ADP‐ribose) glycohydrolase ARH3 (ADPRHL2)), proteins involved in the *Wnt* signaling pathway were most significantly enriched (Supporting Information Figure 12). Indeed, recent studies link Tankyrase‐dependent ADP‐ribosylation to the activation of the Wnt pathway.^[30]^

When H_2_O_2_‐derived oxidative stress was imposed, ADP‐ribosylation increased significantly as reported before.^[25]^ We found that proteins located in the nucleus and involved in DNA repair and replication were mainly enriched by our approach (Figure 5C). Besides ARTD10 (PARP 10), ARTD8 (PARP 14) and ARTD5 (Tankyrase 1) we identified the two major ADP‐ribose transferases ARTD1 and ARTD2. The latter has only been identified sporadically in earlier proteomic studies.[^15^, ^31^] Also major players in DNA repair and replication (e.g., APEX1, FEN1, LIG3, PCNA, PNKP, RFC1, TOP1, TOP2b, XRCC1, XPC) were identified. Overall, our proteomic study corroborates the established model of the role of ADP‐ribosylation stimulated by oxidative stress.^[25]^ Also enriched in this cluster of proteins were those known to be involved in protein sumoylation (Figure 5C) strengthening the notion of a cross‐talk of both PTMs.^[32]^ These proteins include XPC, ARTD1, ARTD2, PCNA, SUMO2, SUMO4, SMC3, TOP1, TOP2 A and TOP2B.

We also investigated HeLa cells in their response to treatment with olaparib, an approved drug that is known to inhibit the major drivers of protein PARylation namely, ARTD1 and ARTD2.^[12b]^ Earlier studies that were based on affinity enrichment by a macrodomain indicate that ADP‐ribosylation was indeed inhibited, since significantly reduced numbers of proteins were enriched upon treatment of olaparib even in the presence of H_2_O_2_‐based oxidative stress.^[31]^ In contrast, we found ADP‐ribosylated proteins involved in DNA repair and replication were rather depleted (cluster 1, Figure 5) but instead proteins were significantly enriched that are involved in RNA‐related processes (cluster 2, Figure 5). In fact, more proteins with a primary involvement in RNA processing were enriched when olaparib was applied in the presence of H_2_O_2_‐based oxidative stress than in the absence of the drug (Figure 5C). Moreover, we found that even in the absence of any H_2_O_2_‐based oxidative stress, olaparib is triggering ADP‐ribosylation, as evidenced by a significant number of enriched proteins (Figure 5). This may be explained by the fact that the major consumers of NAD^+^ is ARTD1.^[21]^ Therefore, inhibiting this enzyme allows other less abundant ARTDs to have access to the NAD^+^ pool leading to a different protein target pattern. Also, olaparib's inhibitory effect of ARTD1 and ARTD2 results from to different modes of action; it blocks the active side in a competitive manner and also increases the affinity of ARTD1 to DNA, e.g., on DNA strand breaks, Okazaki‐fragment‐intermediates, and topoisomerase 1 (TOP1)‐processed ribonucleotides.[^12a^, ^33^] Thus, inhibition of ARTD1 and 2 under basal conditions may increases endogenous DNA damage that will impair gene expression and follow‐up processes like RNA processing and ribosome biogenesis. In fact, recent evidence points towards the involvement of ADP‐ribosylation events in key regulation of protein biogenesis leading from transcription, ribosome biogenesis to mRNA processing and translation.^[34]^

Indeed, we found that under conditions in which the two major nuclear ADP‐ribosylators (ARTD1 and 2) are inhibited, mainly proteins that are involved in these processes are enriched (Figure 5C). This indicates that ADP‐ribosylation events in these processes also occur independently from the catalytic activity of ARTD1 and 2. Since these effects have not been reported before, we attribute this finding to the sensitivity of our approach. Once attached to the protein either directly in MARylation events or within as PAR chain, enrichment relies on the very strong non‐covalent binding of the introduced DTB‐modification to streptavidin and its mild elution by biotin. The superior sensitivity of the herein applied DTB‐streptavidin interaction might be causative for these findings.

## Conclusion

Overall, this study demonstrates that the utility of the newly developed NAD^+^ analogs in combination with our mild and effective cell delivery protocol enables imaging of PAR in living cells and to analyze the cellular adaptation by protein ADP‐ribosylation as a consequence of environmental changes such as H_2_O_2_‐induced oxidative stress or the effect of drugs such as olaparib. Our findings are therefore paving the way for further functional and clinical studies of the ADP‐ribosylated proteome in living cells in health and disease.

## Conflict of interest

The authors declare no conflict of interest.

1

## Supporting information

As a service to our authors and readers, this journal provides supporting information supplied by the authors. Such materials are peer reviewed and may be re‐organized for online delivery, but are not copy‐edited or typeset. Technical support issues arising from supporting information (other than missing files) should be addressed to the authors.

Supporting Information

Supporting Information

## Data Availability

The data that support the findings of this study are available in the supplementary material of this article.
