# Supplementary material for: Profiling of the ADP‐Ribosylome in Living Cells
Source: Angew Chem Int Ed Engl. 2022 Mar 9;61(18):e202200977. doi: 10.1002/anie.202200977 (PMC9315028; doi:10.1002/anie.202200977)
Supplement: Supplementary file 1 — Supporting Information [file ANIE-61-0-s001.pdf]

Angewandte Chemie
Eine Zeitschrift der Gesellschaft Deutscher Chemiker

Supporting Information

# Profiling of the ADP-Ribosylome in Living Cells

*M. Lehner, S. Rieth, E. Höllmüller, D. Spliesgar, B. Mertes, F. Stengel, A. Marx**

# Supplementary Information

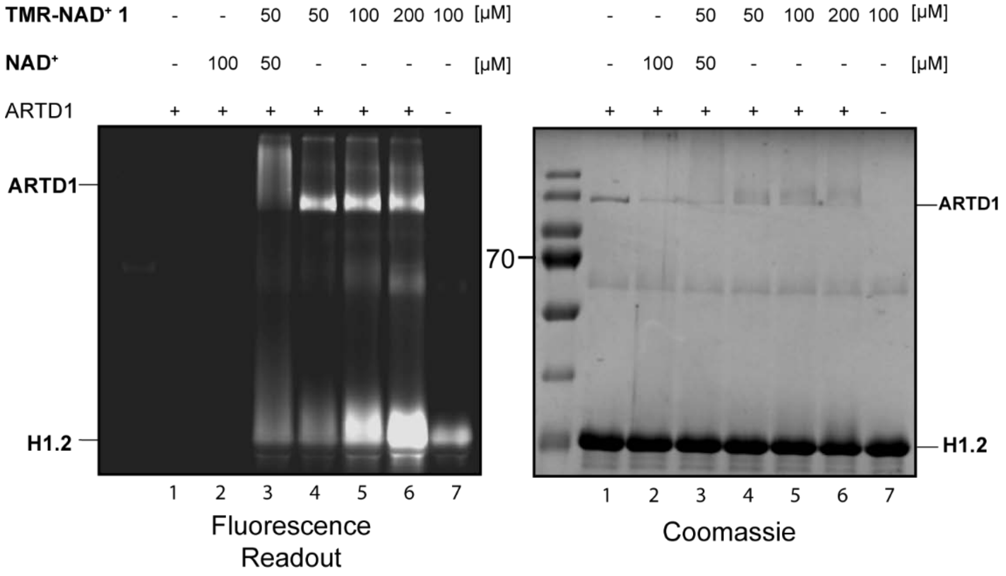


**Supplementary Figure 1**: **SDS-Page analysis of trans-ADP-ribosylation of H1.2 with ARTD1 using TMR-NAD$^+$ 1.** Reactions were performed with **TMR-NAD$^+$ 1.** (lane 4, 5 ,6) and with a 1:1 ration of natural NAD$^+$ and **TMR-NAD$^+$ 1** (lane 3). Controls were performed, without NAD$^+$, with natural. NAD$^+$ (lane 1 and 2) and without enzyme (lane 7) The applied concentrations are indicated above the respective lanes. Unspecific site staining of H1.2 is observed in lane 7 resulting from non-catalytically addition of **TMR-NAD$^+$-1** to the protein.

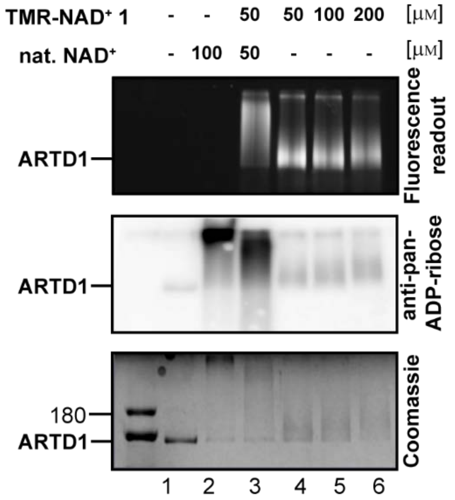


**Supplementary Figure 2: SDS PAGE analyses and Western blot analysis of auto-ADP-ribosylation of ARTD-1 using TMR-NAD $^+$ 1 as substrate.**. Reactions were performed using **TMR-NAD$^+$ 1.** (lane 4, 5 ,6) and a 1:1 ration of natural NAD$^+$and **TMR-NAD$^+$ 1** (lane 3). Controls were performed without NAD$^+$ and with natural. NAD$^+$ (lane 1 and 2). The applied concentrations are indicated above the respective lanes.

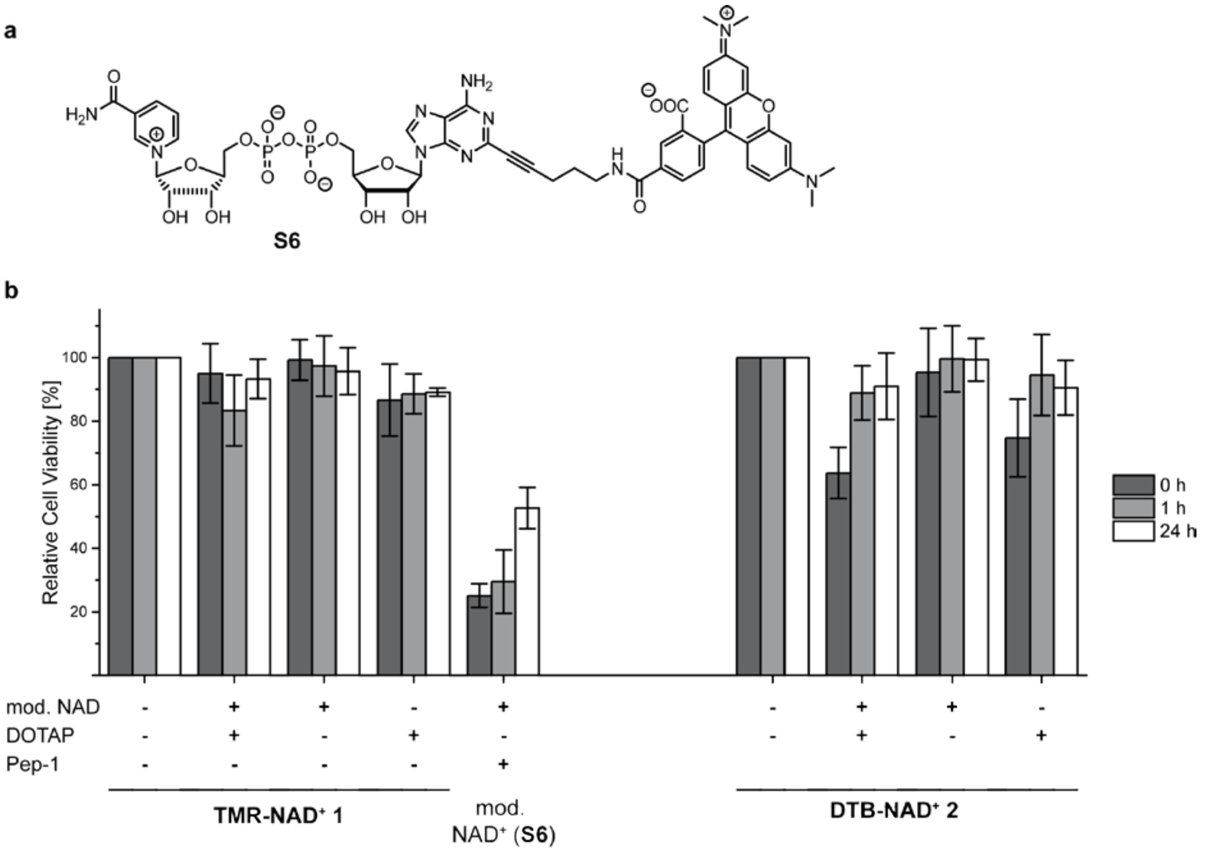


**Supplementary Figure 3**: **Cell viability for various transfection methods. (a)** Structure of TMR-NAD$^+$ analog **S6** recently published by us. **(b)** Plot showing the cell viability of HeLa cells in the presence of the developed transfection method using **TMR-NAD$^+$ 1** and **DTB-NAD$^+$ 2** in comparison to the previously published method using NAD$^+$ (**S6**) and carrier peptide Pep1.[1] HeLa cells (5000 cells per well) were seeded in a 96-well plate and the respective transfection methods were used to transfect mod. NAD$^+$. The cell viabilities in the presence of mod. NAD$^+$ are reported relative to cell viability of HeLa cells without treatment as a control.

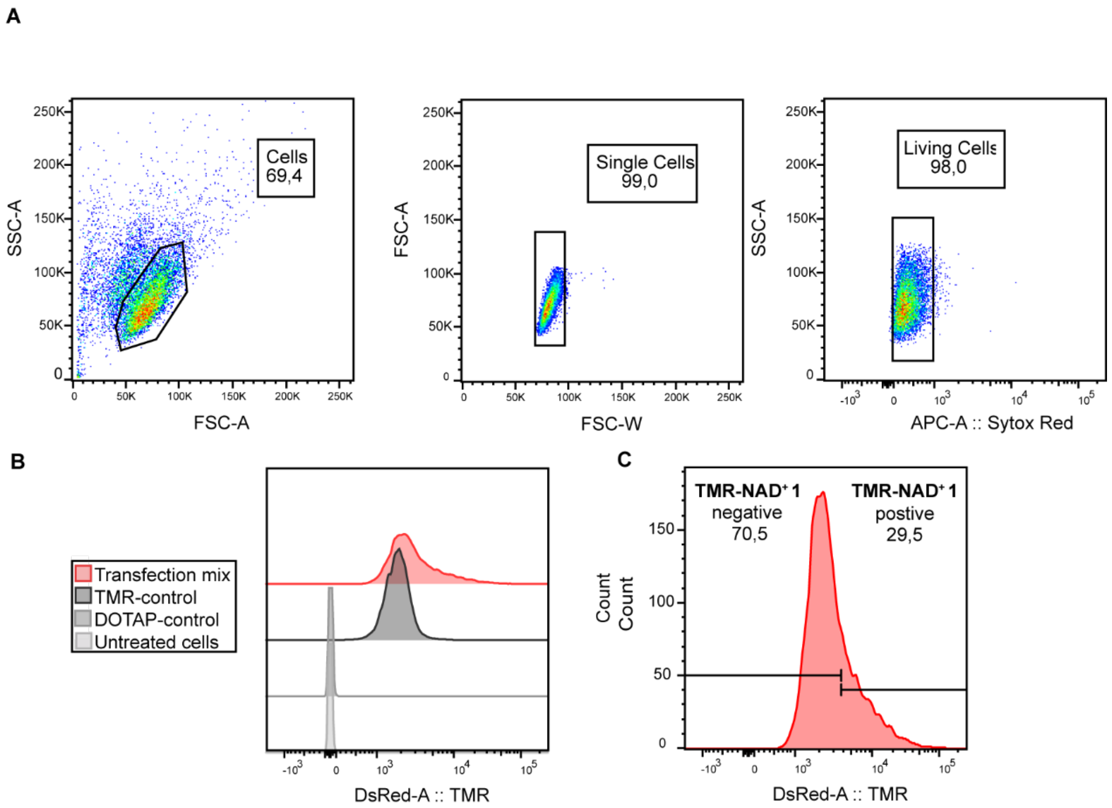


**Supplementary Figure 4: Investigation of the transfection efficiency of TMR-NAD+ 1. A)** Gating strategy of cells with exclusion of duplets via FSC-W / FSC-A parameters and live / dead cell discrimination by SytoxRed labeling. Numbers indicate percentages of the respective population. **B)** Fluorescence intensity of TMR gated on live cells. Representative fluorescence intensity of transfected cells is depicted in red (Transfection-mix), cells treated with only **TMR-NAD+1** (TMR-control, dark grey), Untreated cells (light grey) and cells treated only with DOTAP (DOTAP-control, light grey), were used as controls to identify background fluorescent intensity and cut-off for **TMR-NAD+1**-positive cells. **C)** Gating strategy to determine **TMR-NAD+ 1** positive cells. Numbers indicate the percentage of the representative population.

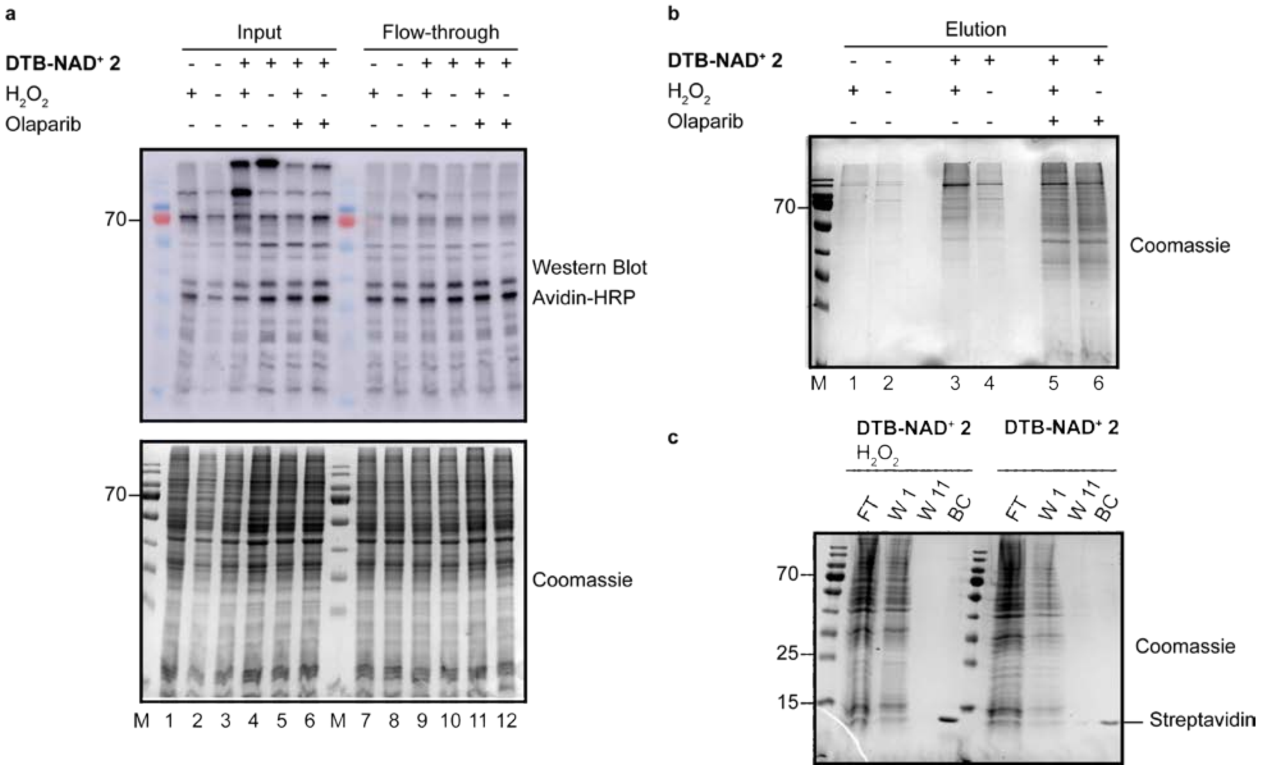


**Supplementary Figure 5**: **SDS-Gel and western blot analysis of the affinity enrichment procedure using DTB-NAD$^+$ 2**. After transfection and cell lysis, the cell lysate was incubated with Streptavidin agarose beads. The flow-through (FT) was separated and the agarose beads were washed several times (W1/ W11). Bound proteins were eluted with biotin and separated by SDS-PAGE. After elution the beads were incubated with 1x loading dye at 95°C for 15 minutes as bead control (BC). **(a)** Cell lysis before (input) and after (flow-through) incubation with streptavidin-agarose beads. **(b)** Elution fractions of the affinity enrichment after PARG treatment. **(c)** Flow-through (FT), wash1 (W1), wash11 (W11) and bead control (BC) of the affinity enrichment assay.

**Supplementary Figure 6: Workflow of affinity enrichment approach.** Identification of endogenous ADP-ribosylated targets in human cell culture by affinity-based proteomic profiling. Schematic workflow of the affinity-based enrichment strategy using a desthiobiotin labeled NAD$^+$ analog

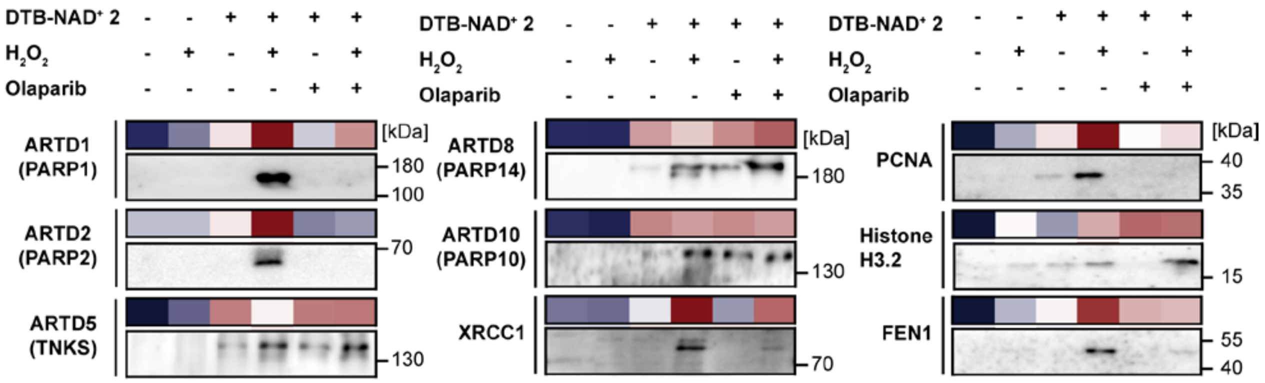


**Supplementary Figure 6 Confirmation of ADP-ribosylated proteins using DTB-NAD$^+$ 2.** Confirmation of ADP-ribosylated targets by western blot analysis. Elution fractions of the affinity enrichment assay were subjected to western blot analysis with specific antibodies for the respective protein and compared to the LC-MS/MS results depicted in the heatmap enrichment pattern.

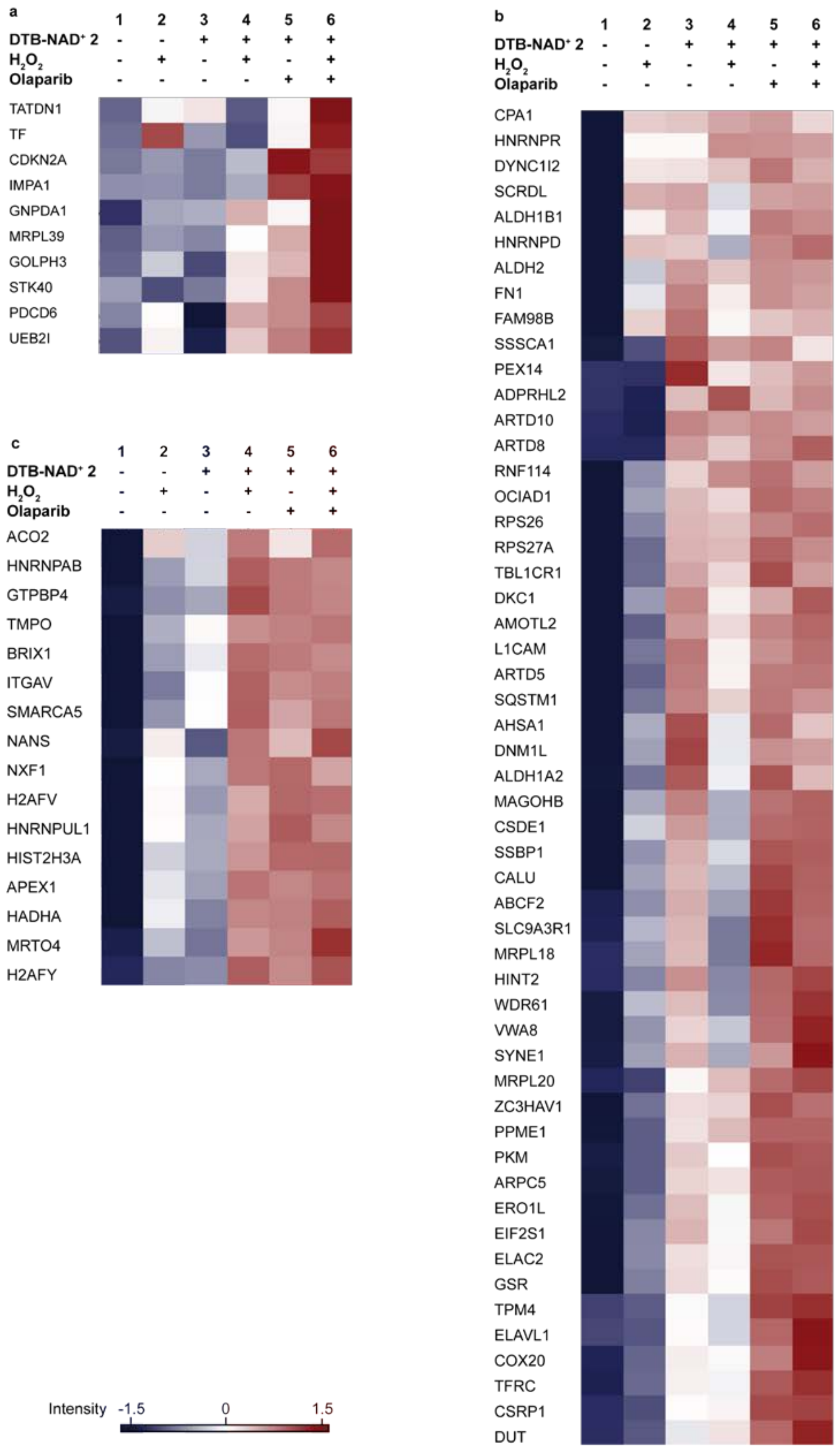


**Supplementary Figure 7: ADP-ribosylated targets from affinity enrichment experiments.** Heatmap of group 1 cluster 2 **(a)**, group 2 cluster 2 **(b)** and group 3 cluster 2 **(c).** For groups and clusters see **Figure 5** of the manuscript.

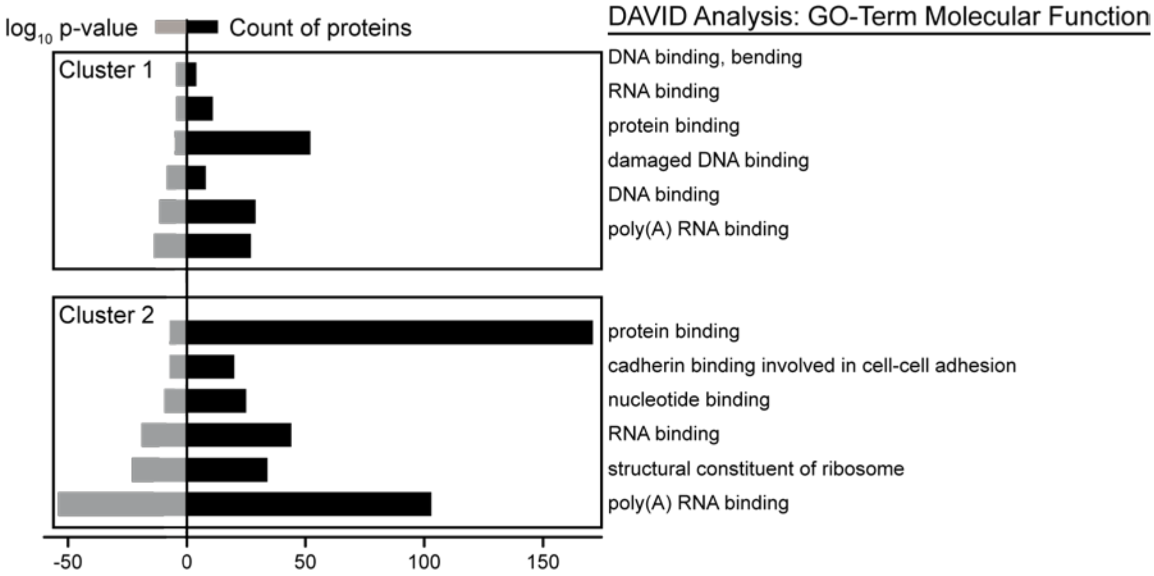


**Supplementary Figure 8: GO-term analysis (Molecular Function)** Proteins significantly enriched in ANOVA analysis were classified according to Molecular Function using DAVID[2] (see **Figure 5A** of the manuscript) (complete dataset see excel sheet). The significance of the enrichment is revealed as a function of the p-value, which indicates whether a biological process is significantly higher than random expectations.

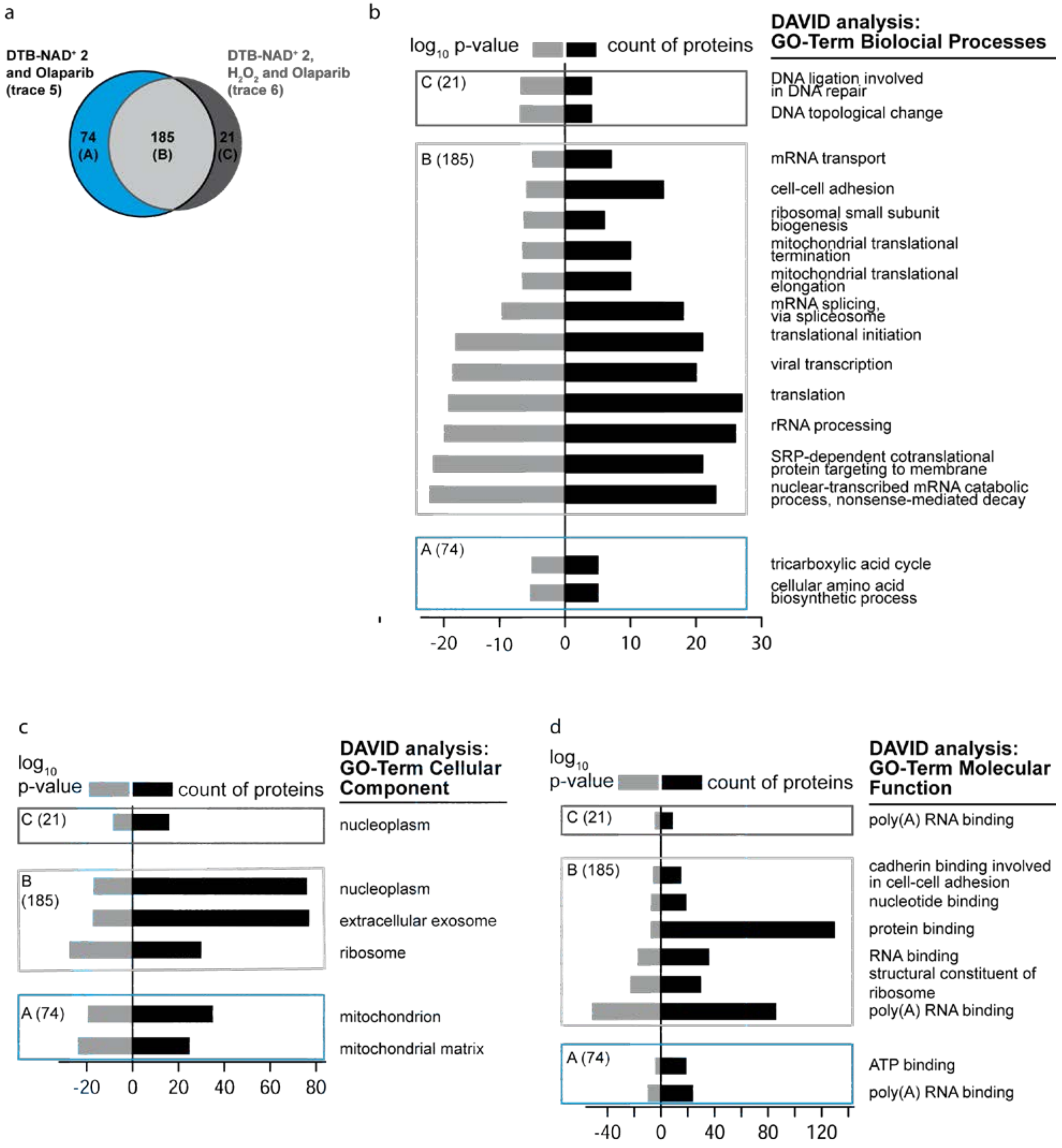


**Supplementary Figure 9**: **GO-term analysis of ADP-ribosylated proteins after olaparib treatment** Proteins significantly enriched in ANOVA analysis in trace 5 and 6 of **Figure 5A** of the manuscript were classified according to Biological Process, Cellular Component and Molecular Function (using DAVID).[2] The significance of the enrichment is revealed as a function of the p-value, which indicates whether a biological process is significantly higher than random expectations.

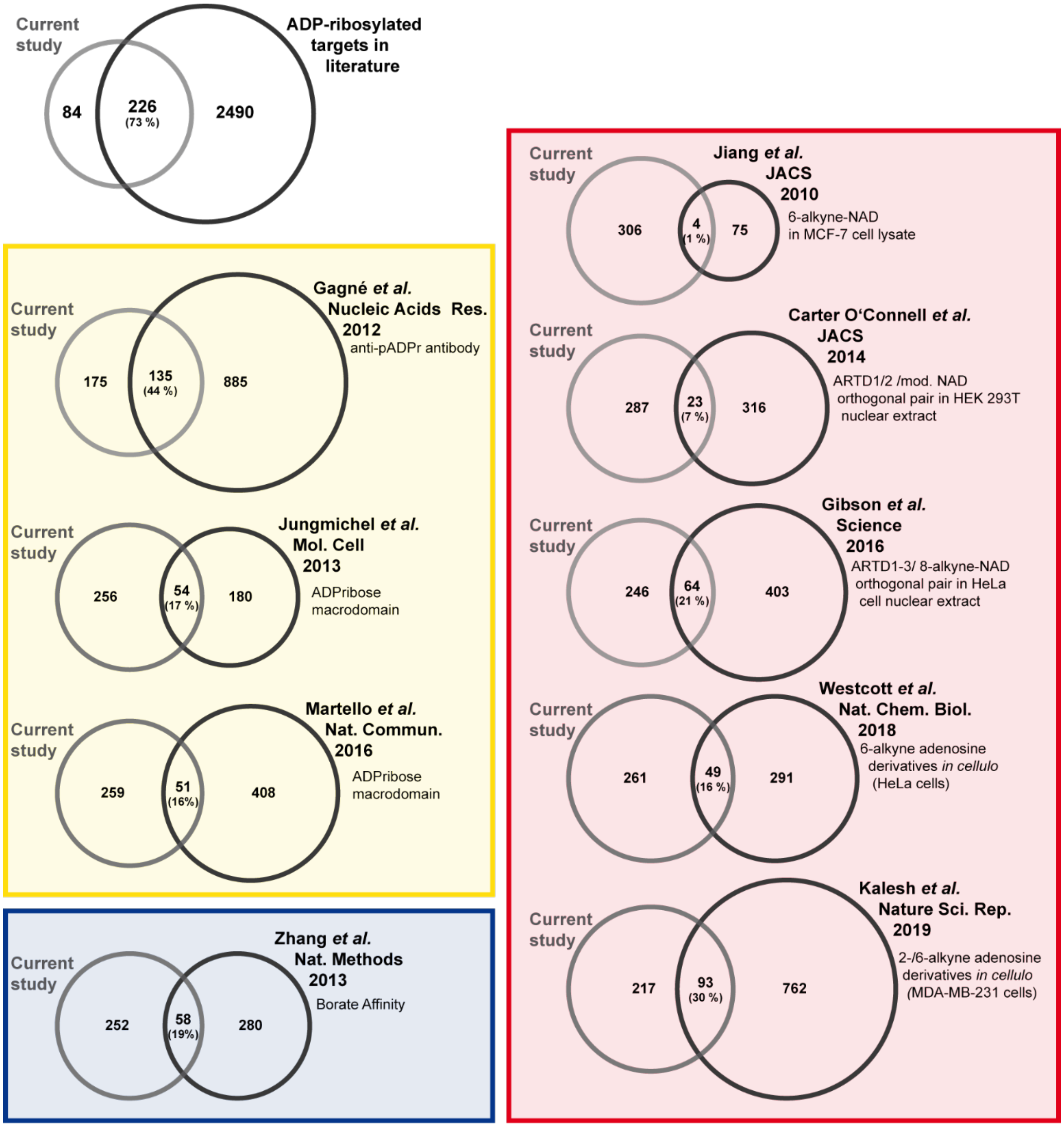


**Supplementary Figure 11: Overlap of ADP-ribosylated target proteins with target proteins reported in the literature**. Venn diagrams show overlap between target proteins identified in this study with all known ADP-ribosylated target proteins in literature, with target proteins enriched with ADP-ribose macrodomains or –antibodies (yellow)[3-5] with target proteins enriched by borate affinity (blue)[6] and with target proteins enriched with nucleoside/nucleotide derivatives (red).[7-11,]

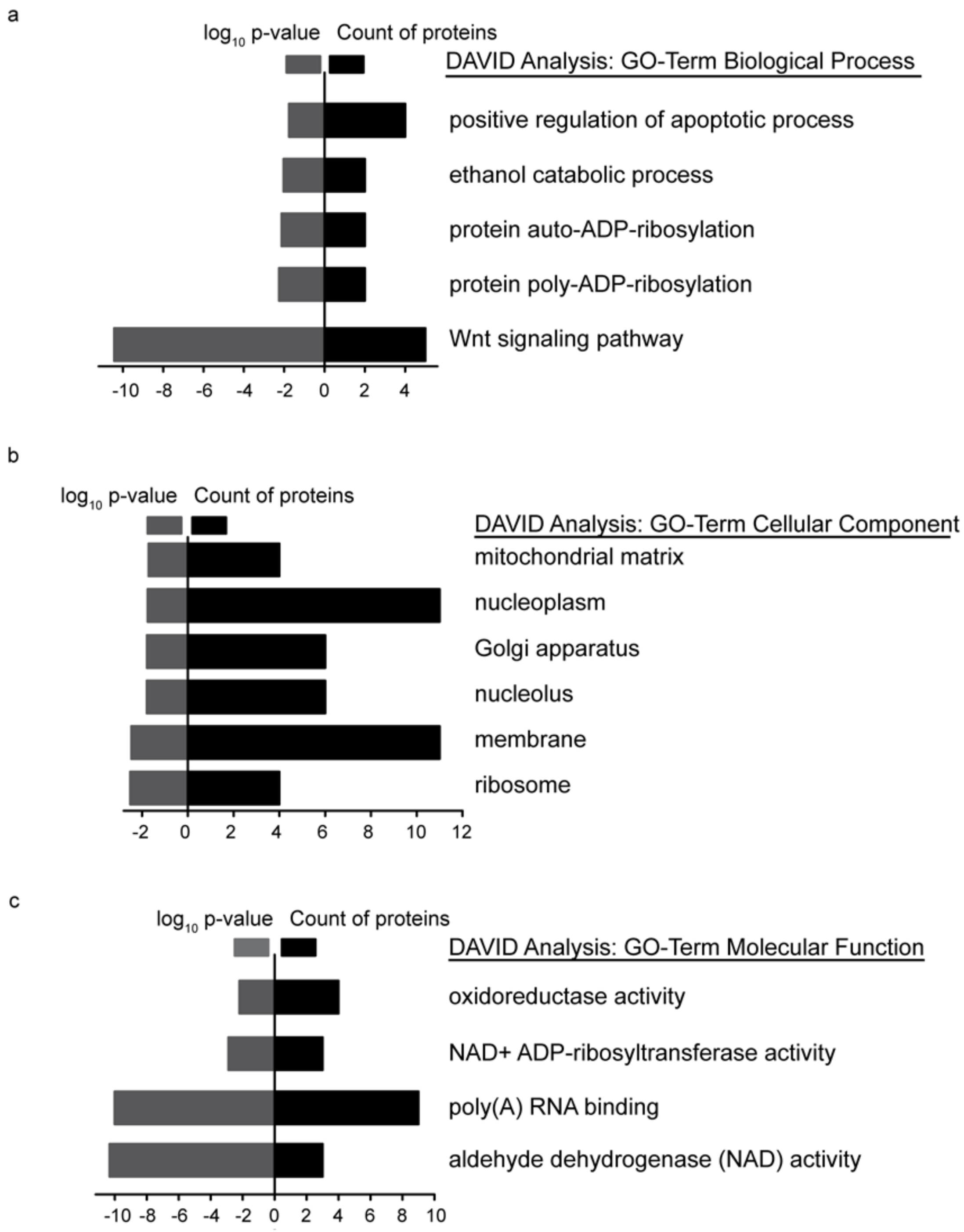


**Supplementary Figur 12: GO-term analysis of ADP-ribosylated proteins**: Proteins significantly enriched in ANOVA analysis in trace 3 was classified according to Biological Process, Cellular Component and Molecular Function (using DAVID). The significance of the enrichment is revealed as a function of the p-value, which indicates whether a biological process is significantly higher than random expectations.

## Synthesis Section

General

All reactions were conducted using standard laboratory techniques. If necessary, due to oxygen or water sensitivity, reactions were performed under the exclusion of air or moisture. In that case dry solvents (VWR) were used. Otherwise, solvents were p.A., absolute or HPLC grade. Commercially available chemicals and solvents were purchased from TCI Chemicals, Sigma-Aldrich/Merck, Acros Organics, Carbosynth, Carl Roth, VWR, BonTac and ABCR. If not denoted differently, they were used without further purification. For chromatography, solvents were p.A., absolute or HPLC grade, technical solvents were distilled before use. For the purification with the HPLC and ion exchange chromatography, water from a combined reverse osmosis/ultrapure water system (Milli-Q) was used.

Flash chromatography was performed under increased pressure (0.3 bar) using silica gel (60 M, pore size 40-63 µm, *Macherey-Nagel*) as the column stationary phase and a step gradient of different solvent systems as the mobile phase. The solvents are specified in the experimental procedures and are quoted as volume (v/v) ratios.

Anion exchange chromatography was performed on a ÄktaPurifier (*GE Healthcare Life Sciences*) at 4 °C. For monophosphate derivatives, a DEAE Sephadex™ A-25 (GE Healthcare Bio-Science AB) column was used with a linear gradient (0.1 – 1 M) of TEAB buffer (pH 7.5) and for $NAD^+$-derivatives a Q Sepharose Fast Flow (GE Healthcare Bio-Science AB) column with a linear gradient of Milli-Q water/triethylammonium bicarbonate (TEAB) buffer (1 M, pH 7.5).

Medium pressure liquid chromatography (MPLC) was performed on a PrepChrom C-700 (*Büchi*) instrument using SVF D22 RP-18 columns (*Götec Labortechnik*) with a linear gradient of Milli-Q water/Acetonitrile or 50 mM TEAB/Acetonitrile. The solvents are specified in the experimental procedures and are stated as volume (v/v) ratios.

High performance liquid chromatography (HPLC) was performed using Shimadzu systems equipped with LC 20AP pumps and SPD M20A PDA detectors. For preparative separations, a 250/21 NUCLEODUR® $C_{18}$ HTec (5 µm, flow rate 15 mL/min) column (*Macherey-Nagel*) or a 250/16 NUCLEODUR® $C_{18}$ HTec (5µm, flow rate 8 mL/min) column (*Macherey-Nagel*) were used. The gradient was adjusted to the respective separation problem and is specified in the experimental procedures. All compounds were obtained as their triethylammonium salts after purification via HPLC.

Nuclear Magnetic Resonance (NMR) spectra were recorded on a Bruker Avance III 400 MHz or 600 MHz spectrometers at room temperature (r.t.). All samples were dissolved in deuterated solvents ($D_2O$) and the spectra were calibrated to the internal standards of the deuterated solvents ($D_2O$: δ($^1$H) 4.79 ppm) or using $^1$H spectrum as absolute reference (for $^{13}$C and $^{31}$P in $D_2O$). Chemical shifts (δ) are given in parts per million (ppm) and coupling constants in Hertz (Hz). For signal assignment two dimensional spectra (HSQC, HMBC, COSY, $^{31}$P-HMBC) were used. NMR spectra were evaluated with MestReNova 12.0.4 (*MestReLab Research S.L.*) and the data were reported to the following convention: chemical shift δ in ppm (multiplicity, coupling constant J in Hz, number of protons/carbons/phosphors and position of the protons/carbons/phosphors in molecule). Multiplicities were specified as s (singlet), d (doublet), t (triplet), q (quadruplet), p (quintet), m (multiplet), br (broad signal) and combinations of given annotations. Counter-ions such as triethylammonium are not assigned in the NMR characterization.

High resolution electro-spray-ionization mass spectra (HR-ESI-MS) were recorded on a *Bruker* Daltronics micrOTOF II in either negative or positive mode with an external Na format calibrant.

Phosphate yields were measured by UV absorption on a NanoDrop UV Visible Spectrophotometers (*Thermo Scientific*) using the published coefficients of the nucleosides or dyes.

Precursor Compounds

The following compounds were commercially obtained:

| **Chemicals** | **Supplier** |
|---|---|
| ***β*-NMN** | Sigma Aldrich/ Merck or BonTac |
| **1,6-diaminohexane** | Sigma Aldrich/ Merck |
| **2-Iodoadenosine** | Sigma Aldrich/ Merck |
| **D-desthiobiotin** | Sigma Aldrich/ Merck |

The following compounds were prepared as previously puplished:

- 5''-Carboxy-tetramethyl rhodamine (TMR)[12]
- TMR NHS ester[13]
- Desthiobiotion NHS ester[14]
- *C*2-(5-(5''-Carboxy-tetramethyl rhodamine)-amidopent-1-yn-1-yl) adenosine nicotinamide dinucleotide (**8**)[1]

## Synthesis of modified AMP analog precursors

**2-Iodoadenosine 5‘-monophosphate (S2)**

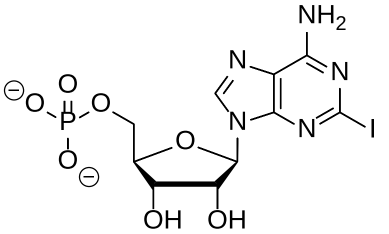


2-Iodoadenosine (**S1**) (1.02 mmol, 400 mg, 1 eq.) were pre-dried overnight *in vacuo*. After dissolving in $PO(OMe)_3$ (5 mL), the solution was cooled to 0 °C. Dry $POCl_3$ (114 µL, 1.2 mmol, 1.2 eq.) was added and the mixture was stirred for 1 to 2 hours on ice until complete conversion, monitored by TLC (DCM/MeOH, 4/1). The reaction was quenched by addition of 0.1 M aqueous TEAB buffer (20 mL). The mixture was extracted with EtOAc (3x 25 mL) and the aqueous phase was concentrated under reduced pressure. The crude product was purified by ion exchange chromatography (DEAE Sepharose, 0.1 M – 1 M TEAB buffer) and by RP-MPLC (C18, 50 mM TEAB/Acetonitrile, 95/5 – 60/40). After lyophilization, the product (**4**) was obtained as its triethylammonium salt (40%, by UV detection).

$^{1}H$ NMR (400 MHz, $D_2O$): $\delta$ 8.40 (s, 1H, H-8), 5.91 (d, $J$ = 5.4 Hz, 1H, H-1‘), 4.65 (t, $J$ = 5.3 Hz, 1H, H2‘), 4.41 (t, $J$ = 4.5 Hz, 1H, H3‘), 4.25 (q, $J$ = 3.7 Hz, 1H, H4‘), 3.92 (m, 2H, H5’a/b) ppm.

$^{13}C$ NMR (100 MHz, $D_2O$): $\delta$ 154.99 (C-6), 149.35 (C-4), 139.83 (C-8), 119.64 (C-2), 118.14 (C-5), 86.99 (C-1‘), 84.63 (C-4‘), 74.69 (C-2‘), 70.55 (C-3‘), 63.39 (C-5‘) ppm.

$^{31}P$ NMR (162 MHz, $D_2O$): $\delta$ 3.80 (s, 1P) ppm.

HR-ESI-MS (m/z) $[M-H]^-$ ($C_{10}H_{12}IN_5O_7P^-$) found 471.9522, calc. 471.9525.

***C*2-(6-aminohexyl)-amino-adenosine 5’-monophosphate (S3)**

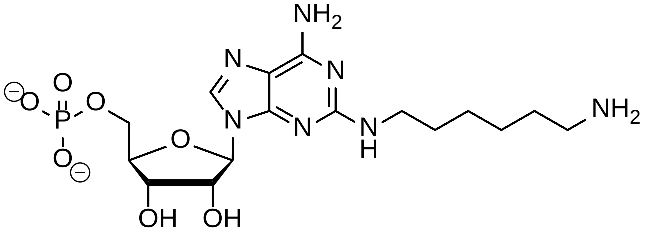


2-Iodoadenosine 5‘-monophosphate (**S2**) (80 µmol, 1 eq.) was dissolved in $H_2O$ (1.5 mL) and 1,6-diaminohexane (18.6 mg, 160 µmol, 2 eq.) and DBU (24 µL, 160 µmol, 2 eq.) were added. The mixture was stirred for 45 min at 150 °C under microwave conditions. The crude product was

purified by RP-HPLC (C18 HTec, 50 mM TEAB/Acetonitrile, 95/5 – 60/40). After lyophilisation, the product (**5**) was obtained as its triehylammonium salt (70%, by UV detection).

$^{1}H$ NMR (400 MHz, $D_2O$) $\delta$ 8.20 (s, 1H, H-8), 6.01 (d, $J$ = 5.7 Hz, 1H, H-1'), 4.85 (t, $J$ = 5.7 Hz, 1H, H-2'), 4.52 (t, $J$ = 4.5 Hz, 1H, H-3'), 4.33 (q, $J$ = 4.0 Hz, 1H, H-4'), 3.99 (m, 2H, H-5'a/b), 3.31 (t, $J$ = 7.0 Hz, 2H, $CH_2$-1*), 2.94 (t, $J$ = 7.6 Hz, 2H, $CH_2$-6*), 1.65 – 1.58 (m, 1H, $CH_2$-5*), 1.56 (t, $J$ = 6.7 Hz, 1H, $CH_2$-2*), 1.35 (m, 4H, $CH_2$-3*, $CH_2$-4*) ppm.

$^{13}C$ NMR (100 MHz, $D_2O$): $\delta$ = 160.14 (C-2), 156.17 (C-6), 151.85 (C-4), 137.66 (C-8), 112.68 (C-5), 86.59 (C-1'), 84.42 (C-4'), 73.78 (C-2'), 70.84 (C-3'), 63.87 (C-5'), 41.14 (C-1*), 39.44 (C-6*), 28.55 (C-2*), 26.72 (C-5*), 25.54 (C-3*), 25.33 (C-4*) ppm.

$^{31}P$ NMR (162 MHz, $D_2O$): $\delta$ = 3.89 (s, 1P) ppm.

HR-ESI-MS (m/z) $[M-H]^-$ ($C_{16}H_{27}N_7O_7P^-$) found 460.1301, calc. 460.1715.

### *C*2-(6-(5''-Carboxy-tetramethyl rhodamine)-amidohexyl)-amino adenosine 5'-mono-phosphate (S4, TMR-AMP)

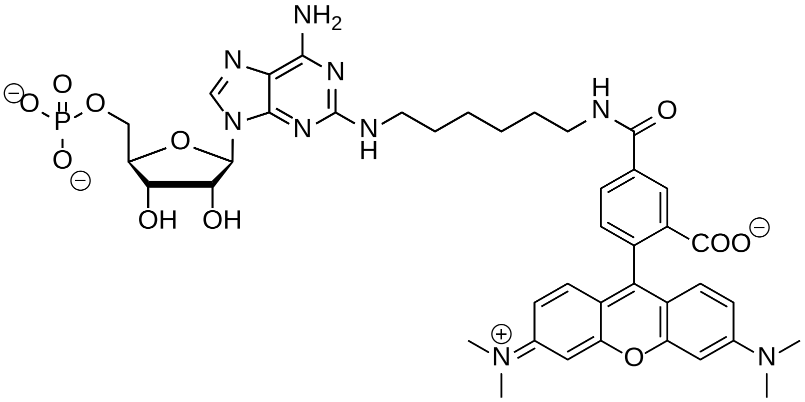


*C*2-(6-Aminohexyl)-amino-adenosine 5'-monophosphate (**S3**) (1 eq.) was dissolved in 0.1 M aqueous $NaHCO_3$ (5 mL). 5-Carboxy-tetramethylrhodamine *N*-succinimidyl ester (TMR NHS ester) (1.5 eq.) was dissolved in DMF (1 mL) and added to the reaction mixture. The pH value was adjusted to pH 8.7 and the reaction mixture was stirred at room temperature overnight. The crude product was concentrated under reduced pressure, dissolved in water and purified via flash chromatography (silica gel, Isopropanol/$H_2O$/$NH_3$ 6/1/1 – 3/1/1) and by RP-HPLC (C18 HTec, 50 mM TEAB buffer/Acetonitrile). After lyophilization, the product (**S4**) was obtained as its triethylammonium salt (70%, by UV detection).

$^{1}H$ NMR (400 MHz, $D_2O$) δ 8.27 (d, *J* = 1.6 Hz, 1H, H-6**), 8.16 (s, 1H, H-8), 7.98 (d, *J* = 7.9 Hz, 1H, H-4**), 7.39 (d, *J* = 7.9 Hz, 1H, H-3**), 7.10 (t, *J* = 9.5 Hz, 2H, H-2*, H-7*), 6.83 – 6.72 (m, 2H, H-1*, H-8*), 6.56 (s, 2H, H-5*, H-4*), 5.90 (d, *J* = 5.7 Hz, 1H, H-1'), 4.68 (t, *J* = 5.6 Hz, 1H, H-2'), 4.48 (t, *J* = 4.4 Hz, 1H, H-3'), 4.24 (m, 1H, H-4'), 3.98 (m, 2H, H-5'a/b), 3.45 (t, *J* = 6.7 Hz, 2H, -$CH_2$-NH-), 3.14 (m, 12 H, 2x $(CH_3)_2$N-), 1.65 (m, 2H, -$CH_2$-), 1.52 (m, 2H, -$CH_2$-), 1.39 (m, 4H, 2x –$CH_2$-) ppm.

$^{13}C$ NMR (100 MHz, $D_2O$): *δ* 172.40 ($COO^-$), 168.35(NHCO), 159.48 (C-10*), 158.44 (C-2), 156.76 (C-9*, C-11*), 156.50 (C-3*, C-6*), 155.50 (C-6), 151.54 (C-4), 141.09 (C-1** or C-5**), 136.90 (C-8), 135.26 (C-1** or C-5**), 134.28 (C-2**), 130.64 (C-2*, C-7*), 130.38 (C-3**), 127.95 (C-4**, C-6**), 113.53 (C-1*, C-8*), 112.63 (C-5), 112.21 (C-12*, C-13*), 96.03 (C-4*, C-5*), 86.17 (C-1'), 84.39 (C-4'), 74.44 (C-2'), 70.87 (C-3'), 63.68 (C-5'), 41.46 (-$CH_2$-), 41.43 (-$CH_2$-), 40.12 (2x $N(CH_3)_2$), 28.87 (-$CH_2$-), 28.76 (-$CH_2$-), 26.37 (-$CH_2$-), 26.29 (-$CH_2$-) ppm.

$^{31}P$ NMR (162 MHz, $D_2O$): *δ* 3.94 (s, 1P) ppm.

HR-ESI-MS (m/z) $[M-H]^-$ ($C_{41}H_{47}N_9O_{11}P^-$) found 872.2539, calc. 872.3138.

**_C_2-(6-(desthiobiotin)-amidohexyl)-amino adenosine 5'-mono-phosphate (S5, DTB-AMP)**

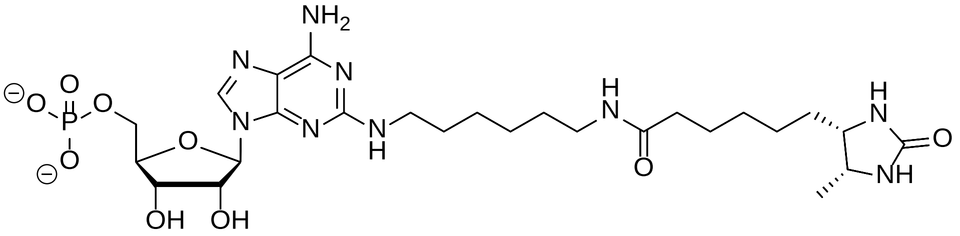


Compound **S3** (1 eq.) was dissolved in 0.1 M aqueous $NaHCO_3$ (5 mL), desthiobiotin *N*-succinimidyl ester (DTB NHS ester) (1.5 eq.) was dissolved in DMF (1 mL) and added to the solution. The pH value was adjusted to pH 8.7 and the reaction mixture was stirred at RT overnight. The crude product was concentrated under reduced pressure, dissolved in water and purified by RP-HPLC (C18 HTec, 50 mM TEAB buffer/acetonitrile). After lyophilisation, the product was obtained as its triethylammonium salt (62 %, by UV detection).

$^{1}H$ NMR (400 MHz, $D_2O$) δ 8.10 (s, 1H, H-8), 5.87 (d, *J* = 5.9 Hz, 1H, H-1'), 4.67 – 4.61 (m, 1H, H-2'), 4.38 (t, *J* = 4.3 Hz, 1H, H-3'), 4.18 (q, *J* = 3.8 Hz, 1H, H-4'), 3.86 (m, 2H, H-5' a/b), 3.65 (p, *J* = 6.7 Hz, 1H), 3.50 (q, *J* = 7.2 Hz, 1H), 3.17 (t, *J* = 7.0 Hz, 2H), 3.04 (t, *J* = 6.7 Hz, 2H), 2.07 (t, *J* = 7.2 Hz, 2H), 1.43 (t, *J* = 6.9 Hz, 4H), 1.35 (d, *J* = 6.2 Hz, 1H), 1.22 (dd, *J* = 14.1, 6.2 Hz, 4H), 0.88 (d, *J* = 6.5 Hz, 3H) ppm.

$^{13}C$ NMR (100 MHz, $D_2O$): $\delta$ 176.40 (-NH**C**O-), 165.30 (C-9**), 159.84 (C-2), 155.86 (C-6), 151.75 (C-4), 137.16 (C-8), 112.38 (C-5), 86.15 (C-1‘), 84.35 (C-4‘), 74.11 (C-2‘), 70.79 (C-3‘), 63.62 (C-5‘), 55.84 (C-6**), 51.33 (C-7**), 41.25 (C-1*), 39.09 (C-6*), 35.64 (C-1**), 28.73 ($CH_2$-Linker), 28.26 ($CH_2$-Linker), 28.08 ($CH_2$-Linker), 25.86 ($CH_2$-Linker), 25.83 ($CH_2$-Linker), 25.30 ($CH_2$-Linker), 25.27 ($CH_2$-Linker), 14.36 (C-11**) ppm.

$^{31}P$ NMR (162 MHz, $D_2O$) δ 3.82 ppm.

HR-ESI-MS (m/z) $[M-H]^-$ ($C_{26}H_{43}N_9O_9P^-$) found 656.2478, calc. 656.2927.

## Synthesis of **TMR-NAD$^+$ 1** and **DTB-NAD$^+$ 2**

### General procedure for NAD$^+$ synthesis

*β*-NMN (10 mg, 30 µmol, 1.5 eq.) and CDI (16 mg, 50 µmol, 1.7 eq.) were dried *in vacuo* overnight and dissolved in dry DMF (1 mL) and dry $NEt_3$ (8 µL, 60 µmol, 2.0 eq.) was added. The mixture was stirred for four hours at room temperature, dry MeOH (0.1 mL) was added and stirred for additional ten minutes. The mixture was concentrated under reduced pressure and the activated *β*-NMN was co-evaporated with dry DMF (3x 1 mL), dissolved in dry DMF (1 mL) and added to the respective pre-dried adenosine 5‘-monophosphate analog (20 µmol, 1.0 eq.). The reaction mixture was stirred at RT for four to five days. The reaction was quenched by the addition of 0.1 M aqueous TEAB (5 mL), stirred for additional ten minutes and the solvents were removed under reduced pressure. The crude product was purified by ion exchange chromatography (Q Sepharose, $H_2O$/1 M TEAB buffer) and RP-HPLC (C18 HTec, 50 mM TEAB buffer/Acetonitrile 95/5 – 60/40). After lyophilisation, the product was obtained as their triethylammonium salt (by UV detection).

**_C_2-(6-(5’’-Carboxy-tetramethyl rhodamine)-amidohexyl)-amino adenosine nicotinamide dinucleotide (TMR-NAD$^+$ 1)**

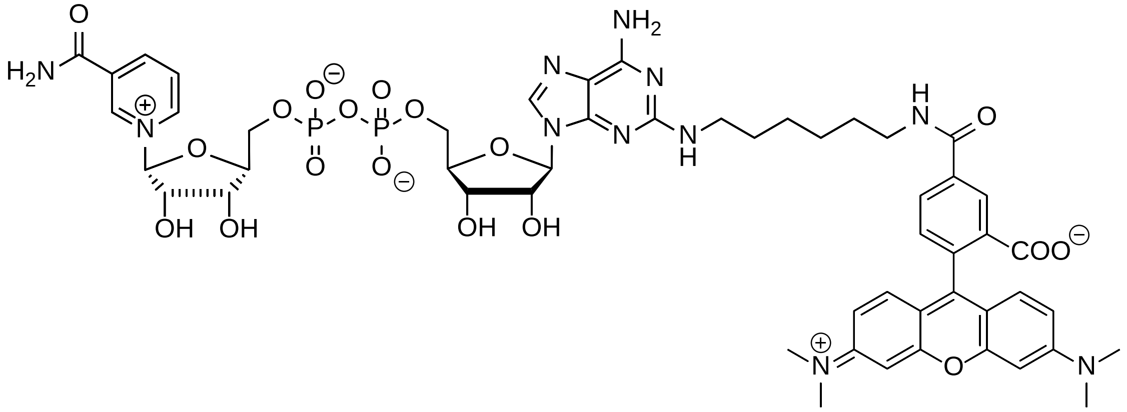

TMR-AMP (**S4**) was coupled with the activated *β*-NMN according to the general method. Following this procedure, the product (**TMR-NAD$^+$ 1**) was obtained as its triethylammonium salt (30%, by UV detection).

$^1$H NMR (400 MHz, $D_2O$) δ 9.19 (s, 1H, H-2’’’), 8.98 (d, *J* = 6.3 Hz, 1H, H-6‘‘‘), 8.71 (d, *J* = 8.1 Hz, 1H, H-4‘‘‘), 8.21 (m, 1H, H-6**), 8.06 (m, 1H, H-5‘‘‘), 7.88 (m, 1H, H-4**), 7.86 (s, 1H, H-8), 7.21 (d, *J* = 8.0 Hz, 1H, H-3**), 6.95 (dd, *J* = 9.3, 4.7 Hz, 2H, H-2*, H-7*), 6.56 (t, *J* = 12.2 Hz, 2H, H-1*, H-8*), 6.32 (m, 2H, H-4*, H-5*H-2*, H-7*), 5.91 (d, *J* = 5.0 Hz, 1H, H-1’’), 5.70 (d, *J* = 5.5 Hz, 1H, H-1’), 4.58 (m, 1H, H-2‘), 4.42 (m, 2H, H-3‘, H-2‘‘), 4.33 (m, 2H, H-3‘‘, H-4‘‘), 4.27 – 4.05 (m, 5H, H-4’, H-5‘ a/b, H-5‘‘ a/b ), 3.31 (m, 2H, NH-$CH_2$-), 2.94 (m, 14H, 2x $N(CH_3)_2$, -$CH_2$-), 1.50 (m, 2H, -$CH_2$-), 1.26 (m, 4H, 2x –$CH_2$-) ppm.

$^{31}$P NMR (162 MHz, $D_2O$) δ -11.33 (d, *J* = 20.1 Hz), -11.68 (d, *J* = 20.3 Hz) ppm.

HR-ESI-MS (m/z) $[M-H]^-$ ($C_{52}H_{61}N_{11}O_{18}P_2^-$) found 1189.2095, calc. 1189.3599.

**_C_2-(6-(desthiobiotin)-amidohexyl)-amino adenosine nicotinamide dinucleotide (DTB-NAD$^+$ 2)**

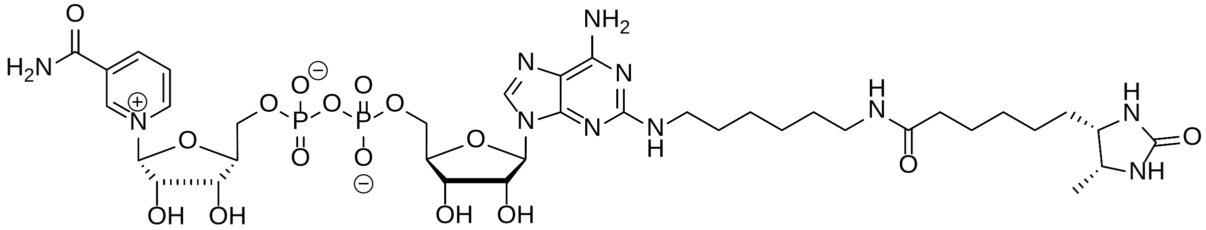

Compound **S5** was coupled with the activated *β*-NMN according to the general method. Following this procedure, the product (**DTB-NAD$^+$ 2**) was obtained as its triethylammonium salt (37%, by UV detection).

$^1$H NMR (400 MHz, $D_2O$) δ 9.32 (s, 1H, H-2'''), 9.10 (d, *J* = 6.3 Hz, 1H, H-6'''), 8.82 (dt, *J* = 8.2, 1.5 Hz, 1H, H-4'''), 8.18 (dd, *J* = 8.1, 6.3 Hz, 1H, H-5'''), 8.02 (s, 1H, H-8), 6.07 (d, *J* = 5.1 Hz, 1H, H-1''), 5.89 (d, *J* = 5.7 Hz, 1H, H-1'), 4.80 (m, 1H, H-2' (below $D_2O$)), 4.55 (t, *J* = 4.6 Hz, 2H, H-3', H-2''), 4.49 – 4.42 (m, 2H, H4', H-3''), 4.36 (m, 2H, H-5'' a/b), 4.26 (m, 3H, H-5' a/b, H-4''), 3.82 – 3.72 (m, 1H, H-7**), 3.63 (q, *J* = 7.3 Hz, 1H, H-6**), 3.26 (d, *J* = 10.8 Hz, 2H, H-1*), 3.20 (m, 2H, H-6* (below TEA), 2.21 (t, *J* = 7.1 Hz, 2H, H-1**), 1.64 – 1.47 (m, 6H, 3x $CH_2$ Linker), 1.39 (d, *J* = 8.0 Hz, 6H, 3x $CH_2$ Linker), 1.27 – 1.11 (m, 4H, 2x $CH_2$ Linker), 1.01 (d, *J* = 6.5 Hz, 3H, $CH_3$-11**) ppm.

$^{31}$P NMR (162 MHz, $D_2O$) δ -11.22 (d, *J* = 20.0 Hz), -11.61 (d, *J* = 21.2 Hz) ppm.

HR-ESI-MS (m/z) $[M-H]^-$ ($C_{37}H_{57}N_{11}O_{16}P_2^-$) found 973.2737, calc. 973.3465.

## Biochemical Methods

<u>Biochemical *in vitro* assays for the acceptance of modified $NAD^+$ analogs</u>

### Auto(ADP-Ribos) ylation of ARTD1

Auto(ADP-ribos) ylation of ARTD1 protocol was adapted from Wang *et al*.[15] (with small modifications). (ADP-ribos) ylation assay was performed in duplicate. For a typical reaction (5 µL), ARTD1 (150 nM) was added on ice to a mixture containing PARylation reaction buffer (Tris-HCl, pH 7.8, 100 mM; $MgCl_2$, 10 mM), dsDNA (5'-GGAATTCC-3', 13 µM) and the respective $NAD^+$ analog (1 mM). The mixture was incubated for 20 min at 37°C and was quenched by the addition of 6x loading dye and denaturation for 5 min at 95 °C. Controls were performed with natural $NAD^+$ (1 mM), without enzyme, with a mixture of natural $NAD^+$ and modified $NAD^+$ (1/1 ratio, $conc._{total}$ 1 mM) and by loading equal amounts of ARTD1. Results were resolved with SDS PAGE analysis (12.5 % separation gel, 35 mA, 35 minutes).

For the fluorescent readout, the gel was placed for 10 minutes in Milli-Q water before taking a picture with a Typhoon FLA 9500 (*GE Healthcare*). For colloidal Coomassie staining, the gel was incubated in fixing solution (methanol, 50 % (v/v); acetic acid, 10 % (v/v)) (2x for 30 minutes) and in the colloidal Coomassie blue staining solution (Roti-Blue® Coomassie stain (5x), 20 % (v/v); methanol, 20 % (v/v)) overnight. Afterwards the gel was placed in colloidal destaining solution (methanol, 25 % (v/v)) for 5-10 minutes and in Milli-Q water for an additional 1 hour before taking a picture with the Amersham Imager 600 (*GE Healthcare*). The image was adjusted in Image Lab™ Version 6.0.1 (*Bio-Rad*).

For western blot analysis the SDS PAGE gel was transferred electrophoretically to a PVDF membrane (0.2 µm, *Invitrogen*). After blocking with milk powder (5 % in TNE-T (TrisHCl, 10 mM; $Na_2EDTA \times 2\ H_2O$, 2.5 mM; NaCl, 50 mM; Tween20; 0.1 %)) for 1 hour at room temperature and washing with TNE-T buffer, the membrane was incubated with ExtraAvidine-HRP (*Sigma Aldrich*; 1:10,0000 in TNE-T buffer) for 1 hour at room temperature. After additional washing (3x TNE-T buffer, 5-10 min), the blot was developed with Pierce™ ECL Western Blotting Substrate (*Thermo Fisher*) in a 1:1 ratio and exposing with Amersham Imager 600 (*GE Healthcare*). Afterwards the membrane was stripped with a stripping buffer (Guanidine HCl, 6 M; TrisHCl, pH 7.5, 20 mM; Mercaptoethanol, 0.1 M; NP-40, 2 %) for 5 minutes two times and washed several times with TNE-T buffer. The membrane was blocked with milk powder (5 % in TNE-T) for 1 hour at room temperature and after additional washing (3x TNE-T buffer, 5-10 min) the membrane was incubated with α-PAR (10-H) (1:3000 in TNE-T) overnight at 4 °C. After several washing steps (3x TNE-T buffer, 5-10 min), the membrane was incubated with an α-mouse secondary antibody

(*Dianova*; 1:5000 in TNE-T buffer) for 1 hour at room temperature. After additional washing (3x TNE-T buffer, 5-10 min), the blot was developed as indicated above. The image was adjusted in Image Lab$^{TM}$ Version 6.0.1 (*Bio-Rad*).

### Auto(ADP-Ribos) ylation of ARTD2

For a typical reaction (5 µL), ARTD2 (150 nM) was added on ice to a mixture containing calf thymus DNA (10 ng/µL), PARylating reaction buffer (Tris-HCl, pH 7.8, 100 mM; $MgCl_2$, 10 mM) and **DTB-NAD$^+$ 2** (50 µM, 100 µM, 200 µM). The mixture was incubated for 1 h at 30°C. The reaction was quenched by the addition of 6x loading dye and denaturation (95 °C for 5 min). Controls were performed with without NAD$^+$, natural NAD$^+$, and with a mixture of natural and modified NAD$^+$ (1/1 ratio, conc.$_{total}$ 200 µM). Results were resolved by SDS-PAGE analysis (12,5 % separation gel, 35 mA, 35 min) and Western blot using with ExtraAvidine-HRP (*Sigma Aldrich*; 1:10,0000 in TNE-T buffer) as indicated above.

### Auto(ADP-Ribos) ylation of ARTD5, ARTD6, ARTD8, ARTD10, ARTD17

For a typical reaction (5 µL). The respective ARTD (150 nm) was added on ice to a mixture containing PARylating reaction buffer (Tris-HCl, pH 7.8, 100 mM; $MgCl_2$, 10 mM) and **DTB-NAD$^+$ 2** (50 µM, 100 µM, 200 µM). The reaction mixture was incubated for a specific time and temperature depending on the enzyme (see Table 1) and quenched by the addition of 6x loading dye and denaturation (95 °C for 5 min). Controls were performed with without NAD$^+$, natural NAD$^+$, and with a mixture of natural and modified NAD$^+$ (1/1 ratio, conc.$_{total}$ 200 µM). Results were resolved by SDS-PAGE analysis (12,5 % separation gel, 35 mA, 35 min) and Western blot ExtraAvidine-HRP (*Sigma Aldrich*; 1:10,0000 in TNE-T buffer) as indicated above.

**Supplementary Table 1** Reaction time and temperature applied in the Auto(ADP-Ribosy)lation assays.

| Name | Incubation time | Incubation temperature |
|---|---|---|
| **ARTD5** | 240 min | 30 °C |
| **ARTD6** | 120 min | 30 °C |
| **ARTD8** | 60 min | 25 °C |
| **ARTD10** | 30 min | 25 °C |
| **ARTD17** | 30 min | 30 °C |

**Trans(ADP-Ribos)ylation of H1.2 with ARTD1**

Trans(ADP-Ribos)yaltion assays were performed in duplicates. For a typical reaction (5 µL), ARTD1 (150 nM) was added on ice to a mixture containing PARylation reaction buffer (Tris-HCl, pH 7.8, 100 mM; $MgCl_2$, 10 mM), dsDNA (5'-GGAATTCC-3', 13 µM) **TMR-NAD$^+$ 1** (50 µM, 100 µM, 200 µM). The mixture was incubated for 20 min at 37°C and was quenched by addition of 6x loading dye and denaturation for 5 min at 95 °C. Controls were performed with natural NAD$^+$ (100 µM,), without NAD$^+$, with a mixture of natural NAD$^+$ and modified NAD$^+$ (1/1 ratio, conc.$_{total}$ 200 µM M) and without enzyme. Results were resolved with SDS PAGE analysis (12.5 % separation gel, 35 mA, 35 minutes and fluorescence read out was performed as indicated above.

**Auto(ADP-Ribos)ylation of ARTD1 with TMR-NAD-1**

Auto(ADP-ribos)ylation assays was performed in duplicate.For a typical reaction (5 µL), ARTD1 (150 nM) was added on ice to a mixture containing PARylation reaction buffer (Tris-HCl, pH 7.8, 100 mM; $MgCl_2$, 10 mM), dsDNA (5'-GGAATTCC-3', 13 µM) and **TMR-NAD$^+$1** (50 µM, 100 µM, 200 µM). The mixture was incubated for 20 min at 37°C and was quenched by the addition of 6× loading dye and denaturation for 5 min at 95 °C. Controls were performed with natural NAD$^+$ (100 µM), without enzyme, with a mixture of natural NAD$^+$ and modified NAD$^+$ (1/1 ratio, conc.$_{total}$ 100 µM) and by loading equal amounts of ARTD1. Results were resolved with SDS PAGE analysis (12.5 % separation gel, 35 mA, 35 minutes) and analyzed via fluorescent readout and Western blot as described above, using anti-pan-ADP-ribose (Merkc,1:5000 in TNE-T buffer) and an α-mouse secondary antibody (*Dianova*; 1:5000 in TNE-T buffer) 1 h at room temperature.

Biochemical *in cellulo* assays

**Transfection of TMR- NAD$^+$ 1 with DOTAP**

HeLa cells were cultured in Dulbecco's Modified Eagle Medium (DMEM) supplemented with 10 % fetal bovine serum (FBS). 24 hours before the experiment, 50,000 cells/cm$^2$ were seeded in 8-well ibiTreat µ-Slides (*ibidi*). A 2x solution of TMR-NAD$^+$ 1 (100 µM) in Opti-MEM and a 2x DOTAP (*N*-[1-(2,3-dioleoyloxy)propyl]-*N*,*N*,*N*-trimethylammonium methyl-sulfate, 30 µM) solution in Opti-MEM were prepared and the solutions were mixed and incubated for 15 min at room temperature to obtain the transfection solution (**TMR-NAD$^+$ 1** (50 µM), DOTAP (15 µM)). The cells were washed once with 1x PBS (pH 7.5) and treated with 200 µL of the transfection solution for 1 hour at 37 °C and 5 % $CO_2$. The cells were washed with 1x PBS (3x 200 µL) and nuclei staining was performed

by Hoechst 33342 (5 µg/mL in Opti-MEM) for 20 min at 37 °C and 5 % $CO_2$. Cells were washed twice with 1x PBS, DMEM (phenol red free, 200 µL) was added and the samples were analyzed by confocal light scanning microscopy.

We also tested Lipofectamine 2000/ 3000/ RNAiMAX (*Invitrogen*), Oligofectamine (*Invitrogen*), Effectene (*Qiagen*), and JetPRIME (*Polyplus*) and obtained inferior results.

### Cell viability of transfected cells

For the cytotoxicity of modified $NAD^+$ analogs on HeLa cells, the CellTiter-Glo® Luminescent Cell Viability Assay (*Promega*) was used according to the instructions provided by the kit manufacturer. HeLa cells (5,000 cells/well) were seeded in 96-well plate (*Sarstedt*) in DMEM supplemented with 10 % FBS without antibiotics 24 hours before experiment. The transfection solution (**TMR-NAD⁺ 1** (50 µM), DOTAP (15 µM) or **DTB-NAD⁺ 2** (100 µM), DOTAP (20 µM)) in Opti-MEM was prepared and incubated for 15 min at room temperature. After washing the cells once with 1x PBS (pH 7.5, 50 µL), the cells were transfected with transfection solution containing respective $NAD^+$ analog and DOTAP and incubated for 1 hour at 37 °C and 5 % $CO_2$. Controls were performed using only mod. $NAD^+$ without DOTAP, only DOTAP or only Opti-MEM. After treatment, the cells were washed with 1x PBS (3x 50 µL) and DMEM supplemented with 10 % FBS without antibiotics (50 µL) was added. The transfected cells were equilibrated at room temperature for 30 min and afterwards CellTiter-Glo® Reagent (50 µL) was added and contents were mixed for 2 min on an orbital shaker and incubated additional 10 min at room temperature. The luminescence signal was recorded on a 1420 Multilabel Counter $Victor^3$ plate reader (*Perkin Elmer*) and the cell viability was normalized to cells incubated with Opti-MEM alone as control.

### Flow cytometry

Hela cells were cultured in Dulbecco's Modified Eagle Medium (DMEM) supplemented with 10% fetal bovine serum (FBS). 24 hours before the experiment 6.5 x $10^4$cells were seeded per well in 24 well-plates (Sarstedt). Cells were treated according to the transfection protocol with **TMR-NAD+-1** (50 µM) and DOTAP (15 µM) (200 µL of the transfection mix per well). After the treatment, the cells were washed with 1x PBS (3× 500 µL), detached with trypsin, pelleted 5 min at 500 g and resuspended in buffer (1x PBS EDTA 1 mM). Dead cells were stained by Sytox Red (1 µM, *Thermofisher Scientific*). Cells were analyzed on a BD LSRII

flow cytometer (*BD Bioscience)*. Sytox Blue and TMR were excited at 405 nm and 561 nm, respectively. Fluorescent signals were detected with a 450/50 bandpass filter (Sytox Red channel) and a 595/20 bandpass filter (TMR channel). Control experiments were performed with untreated cells, DOTAP treated cells and cells treated only with TMR-NAD$^+$. Data analyses were performed with Flow software (FlowJo®)

**PAR staining using TMR-NAD$^+$ 1**

The transfection protocol with **TMR-NAD$^+$ 1** and DOTAP was performed in 8-well ibiTreat µ-Slides (*ibidi*) indicated above. After washing the cells with 1x PBS (3x 200 µL), oxidative stress was induced by $H_2O_2$ treatment (1 mM) in PBS for 10 min at 37 °C and 5 % $CO_2$ to activate ARTD1. As negative control, cells were incubated with ARTD inhibitor olaparib (10 µM) in Opti-MEM before the $H_2O_2$ treatment. After removing the $H_2O_2$ treatment, the cells were washed with 1x PBS (2x 200 µL) and fixed with 4 % para-formaldehyde in 1x PBS (200 µL) for 20 min at room temperature. The PFA treatment was quenched with a $NH_4Cl$ solution (50 mM) and nuclei staining was performed by Hoechst 33342 (5 µg/mL in Opti-MEM) for 30 min at room temperature. Cells were washed twice with 1x PBS, 1x PBS (200 µL) was added and the samples were analyzed by confocal light scanning microscopy.

**Transfection of DTB NAD$^+$ 2 and imaging of PARylated proteins**

HeLa cells were cultured in Dulbecco's Modified Eagle Medium (DMEM) supplemented with 10 % fetal bovine serum (FBS). 24 hours before the experiment, 50,000 cells/cm$^2$ were seeded in 8-well ibiTreat µ-Slides (*ibidi*). A 2x solution of **DTB-NAD$^+$ 2** (200 µM) in Opti-MEM and a 2x DOTAP (*N*-[1-(2,3-dioleoyloxy)propyl]-*N*,*N*,*N*-trimethylammonium methyl-sulfate, 40 µM) solution in Opti-MEM were prepared and afterwards, the solutions were mixed and incubated for 15 min at room temperature to obtain the transfection solution (**DTB-NAD$^+$ 2** (100 µM), DOTAP (20 µM)). The cells were washed once with 1x PBS (pH 7.5, 200 µL) and treated with 200 µL of the transfection solution for 1 hour at 37 °C and 5 % $CO_2$. The cells were washed with 1x PBS (3x 200 µL) and for the activation of ARTD1, an oxidative stress was induced using $H_2O_2$ (1 mM) in 1x PBS (pH 7.5, 200 µL) for 10 min at 37 °C and 5 % $CO_2$. As negative control, cells were incubated with ARTD inhibitor olaparib (10 µM) in Opti-MEM before the $H_2O_2$ treatment. The cells were washed with 1x PBS (200 µL) and fixed by adding 4 % *para*-formaldehyde (PFA) in 1x PBS (200 µL) for 20 min at room temperature. The PFA treatment was quenched with a $NH_4Cl$ solution (50 mM) in 1x PBS (200 µL) for 5 min at room temperature and the cells were permeabilized with Triton X-100 (0.5 %

in 1x PBS, 200 µL) for 20 min at room temperature. The permeabilized cells were washed with BSA (3 % in 1x PBS, 3x 200 µL) and incubated with Cy$^{TM}$5-Streptavidin (*Invitrogen by Thermo Fisher Scientific*, 1:20) and Hoechst 33342 (5 µg/mL) in 1x PBS (200 µL) for 30 min at room temperature. After washing with 1x PBS (3x 200 µL), 1x PBS (200 µL) was added and the samples were analyzed by confocal light scanning microscopy.

**Affinity enrichment of PARylated proteins in human HeLa cells**

HeLa cells were cultured in Dulbecco's Modified Eagle Medium (DMEM) supplemented with 10 % fetal bovine serum (FBS). 24 hours before the experiment, 30,000 cells/cm$^2$ were seeded in 15 cm cell culture dishes (*Sarstedt*). A 2x NAD$^+$ solution of **DTB-NAD$^+$ 2** (200 µM) in Opti-MEM and a 2x DOTAP (*N*-[1-(2,3-dioleoyloxy)propyl]-*N*,*N*,*N*-trimethylammonium methylsulfate, 40 µM) solution in Opti-MEM were prepared and afterwards, the solutions were mixed and incubated for 15 min at room temperature to obtain the transfection solution (**DTB-NAD$^+$ 2**(100 µM), DOTAP (20 µM)). The cells were washed once with 1x PBS (pH 7.5, 10 mL) and treated with 12 mL of the transfection solution for 1 hour at 37 °C and 5 % $CO_2$. The cells were washed with 1x PBS (3x 10 mL) and for the activation of ARTD1, an oxidative stress was induced using $H_2O_2$ (1 mM) in 1x PBS (pH 7.5, 200 µL) for 10 min at 37 °C and 5 % $CO_2$. Controls were performed without $H_2O_2$ treatment, with ARTD inhibitor olaparib, with only $H_2O_2$ treatment and no treatment. Afterwards, the $H_2O_2$ solution was removed and the dishes were placed on ice. Ice-cold lysis buffer (500 µL, Guanidine hydrochloride, 6 M; TrisHCl, pH 8, 50 mM; Na orthovanadate, 2 mM; NaF, 5 mM, glycerol-2-phosphate, 5 mM) was added and after 5-10 min of incubation, the cells were scraped and the suspension was transferred to a 2 mL low-binding tube placed on ice. After sonication (30 pulses, lowest intensity), the supernatant was cleared by centrifugation (14000 g, 30 min, 4 °C).

The samples were diluted to 1.5 mL with buffer (TrisHCl, pH 8, 50 mM; Na orthovanadate, 2 mM; NaF, 5 mM, glycerol-2-phosphate, 5 mM) to obtain a final amount of Gnd-HCl of 2 M and DTT (1 mM) was added. Strep-Agarose beads slurry (70 µL) were equilibrated with buffer (TrisHCl, pH 8, 50 mM; Na orthovanadate, 2 mM; NaF, 5 mM, glycerol-2-phosphate, 5 mM; 3x 35 µL, centrifuged for 90 s at 500 g and rt) and added to each sample. The mixtures were incubated for 1.5 h at room temperature (*Thermo Scientific*). For washing and elution Morbicol "F" columns (*Mo Bi Tec*) with inserted 35 µm filter were used. The columns were equilibrated with buffer (TrisHCl, pH 8, 50 mM; Na orthovanadate, 2 mM; NaF, 5 mM, glycerol-2-phosphate, 5 mM; 2x 100 µL, spin down), placed in a fresh 2 mL tube and afterwards each sample was transferred onto one column and centrifuged to obtain the flow through. The beads on the column were washed with 1 % SDS

in 1x PBS (3x 100 µL, spin down), 4 M urea, 150 mM NaCl and 1 % SDS in 1x PBS (3x 100 µL, centrifugation) and with 50 mM $NH_4HCO_3$ (aq., 5x 100 µL, centrifugation) and every fraction was collected in a new tube. For elution of the PARylated proteins, the beads were incubated with 0.8 mM biotin in 50 mM $NH_4HCO_3$ for 15 min at 37 °C and 950 rpm and afterwards centrifuged in a new low-binding tube (1.5 mL) for 5 s at 500 g and room temperature. This step was repeated twice and the elution fraction of each samples were combined, lyophilized and stored at -20 °C. Samples for SDS PAGE analysis were prepared from each fraction.

**PARG treatment for degradation of PAR**

Elution fractions were re-suspended in PARG reaction buffer ($KH_2PO_4$, 5 mM, pH 7.5; KCl, 5 mM, β-mercaptoethanol, 0.9 mM) and PARG (10 nM) was added. The mixtures were incubated for 4 hours at 37 °C. Afterwards, the reaction was quenched by the addition of 6x loading dye, denaturation for 5 min at 95 °C and resolved by SDS PAGE analysis (12.5 % separation gels, 35 mA, 25 min). Colloidal Coomassie staining and imaging was performed as indicated above.

**In-gel digestion**

In-gel digestion protocol was adapted from Shevchenko et al, 2006 Nature protocols with small modifications.[16] After separation of the elution fractions by SDS-PAGE followed by Coomassie staining, each gel lane was cut into four (4) pieces and destained in $NH_4HCO_3$ (50 mM)/acetonitrile (1/1 ratio, v/v) and washed with $NH_4HCO_3$ (50 mM). Next, disulfide bonds were reduced by the addition of 10 mM DTT in 50 mM $NH_4HCO_3$ (50 mM) for 1 hour at 56 °C, followed by alkylation in 2-chloroacetamide (50 mM) in $NH_4HCO_3$ (50 mM) for 1 hour at r. t. in the dark. After washing in $NH_4HCO_3$ (50 mM)/acetonitrile (1/1 ratio, v/v) and dehydration in acetonitrile, proteins were digested overnight at 37 °C using trypsin (1:50, w/w) (*Promega*). Peptides were extracted in formic acid (5 %)/acetonitrile (1/2 ratio, v/v) and formic acid (5 %)/acetonitrile (2/1 ratio, v/v). The combined extracts were freeze-dried.

Samples were desalted using $ZipTip_{\mu C18}$® Pipette Tips (*Merck Millipore*).

### LC-MS/MS measurements and data analysis

Peptides were analyzed on a QExcactive™ HF Hybrid Quadrupole-Orbitrap™ coupled to an EASY-nLC™ 1200 system (Thermo Scientific). The LC gradient for peptide separation started from 5% acetonitrile, 0.1% formic acid going to 35% acetonitrile, 0.1% formic acid in 45 min, followed by 5 min to 45% acetonitrile, 0.1% formic acid and a washing step at 80% acetonitrile, 0,1% formic acid. The mass spectrometer was operated in data dependent Top20 mode with dynamic exclusion set to 30 s. Full scan MS spectra were acquired at a resolution of 120000 (at m/z 200), scan range 350-1600 m/z with an automatic gain control target value of $3e^6$ and a maximum injection time of 60 ms. Most intense precursors with charge states of 2-6 reaching a minimum automatic gain control target value of $2e^3$ were selected for MS/MS experiments. Normalized collision energy was set to 28%. MS/MS spectra were collected at a resolution of 30000 (at m/z 200), an automatic gain control target value of $1e^5$ and 100 ms maximum injection time. Each of the biological triplicate samples was additionally measured as a technical duplicate.

Raw files were analyzed using MaxQuant (version 1.6.1.0)[17,18] with match between runs and label-free quantification (minimum ratio count 1) enabled. For protein identification, the human reference proteome downloaded from the UniProtKB database (download date: 2018-02-22) and the integrated database of common contaminants were used. Further data processing was performed using Perseus software (version 1.6.1.3)[19]. Identified proteins were filtered for reverse hits, common contaminants and proteins only identified by site. LFQ intensities were log2 transformed, filtered to be detected in at least 5 out of 6 replicates and missing values were imputed from normal distribution (width = 0.3 and shift = 1.8), based on the assumption that these proteins were below the detection limit. Significantly enriched proteins were identified by an ANOVA test (FDR = 0.002, s0 = 2), averaged and normalized by Z-scoring. Finally, enriched proteins were analyzed by hierarchical clustering (Euclidean distance) and plotted as heatmap. Proteins with a minimum Z-score of 0.4 were considered for further analysis in the respective groups.

### Immunoblotting

After the PARG treatment, elution fractions (25 or 50 % of each fraction) were separated by SDS-Gel analysis and transferred electrophoretically to a PVDF membrane (0.2 µm, *Invitrogen*). After blocking with milk powder (5 % in TNE-T (TrisHCl, 10 mM; $Na_2EDTA$ x 2 $H_2O$, 2.5 mM; NaCl, 50 mM; Tween20; 0.1 %)) for 1 hour at room temperature and washing with TNE-T buffer, the membrane was probed overnight with primary antibodies (see Table). The membrane was washed

wit TNE-T buffer (3x, 5 min) and species-specific horseradish peroxidase-conjugated antibodies were added for 1 hour. After additional washing (3x TNE-T buffer, 5-10 min), the membrane was developed with Pierce$^{TM}$ ECL Western Blotting Substrate (*Thermo Fisher*) in a 1:1 ratio and exposing with Amersham Imager 600 (*GE Healthcare*).

**Supplementary Table 2**: List of primary antibodies used for Immunoblotting.

| **Primary Antibody** | | **Supplier** | **Dilution in TNE-T** |
|---|---|---|---|
| **ARTD1/PARP1** | CII10 | | 1:300 |
| **ARTD2/PARP2** | | *Active motife* | 1:500 |
| **ARTD5/TNKS1/PARP5a** | E-10 | *Santa Cruz Biotechnology* | 1:200 |
| **ARTD8/PARP14** | C-1 | *Santa Cruz Biotechnology* | 1:100 |
| **ARTD10/PARP10** | 5H11 | *Santa Cruz Biotechnology* | 1:200 |
| **XRCC1** | | *Enzo Life Sciences* | 1:2000 |
| **PCNA** | | *Antibodies-online* | 1:2000 |
| **Histone H3** | | *Abcam* | 1:2000 |
| **FEN1** | | *Santa Cruz Biotechnology* | 1:1000 |

**Supplementary Table 3**: Horseradish peroxidase conjugated secondary Antibodies used for Immunoblotting.

| **Secondary Antibody** | **Supplier** | **Dilution in TNE-T** |
|---|---|---|
| **Anti-mouse-HRP** | *Dianova* | 1:2000 |
| **Anti-rabbit-HRP** | *Dianova* | 1:1000 |
| **Anti-rat-HRP** | *GE Healthcare* | 1:500 |

## Data availability

The mass spectrometry proteomics data have been deposited to the ProteomeXchange Consortium via the PRIDE[20] partner repository with the dataset identifier PXD020549 (Username: reviewer40609@ebi.ac.uk; Password: xNEgVG6t).

**2-Iodoadenosine 5‘-monophosphate (S2)**

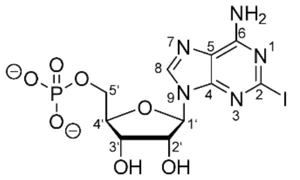

$^{1}H$ NMR (400 MHz, $D_2O$)

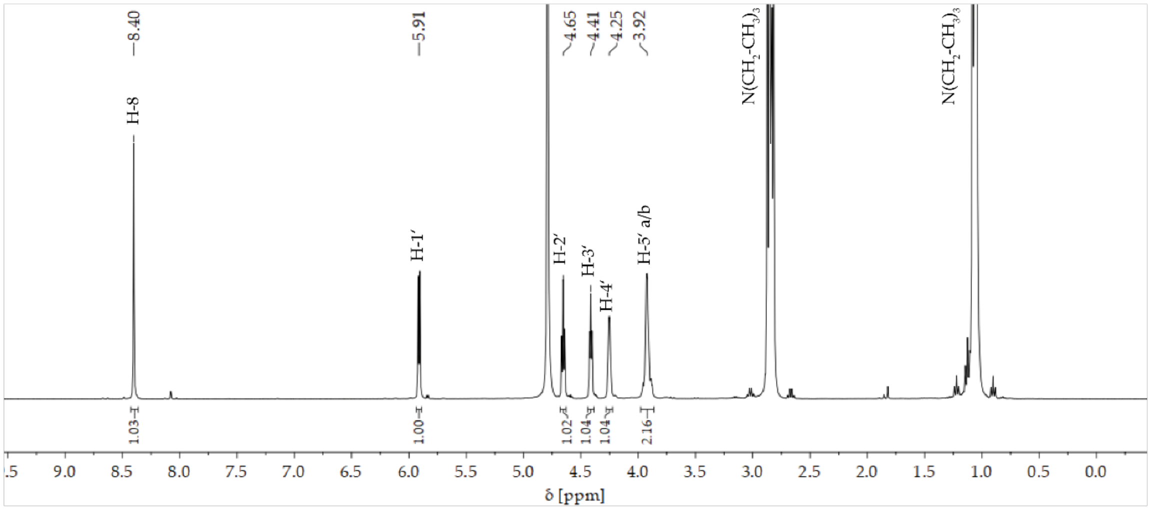


$^{13}C$ NMR (100 MHz, $D_2O$)

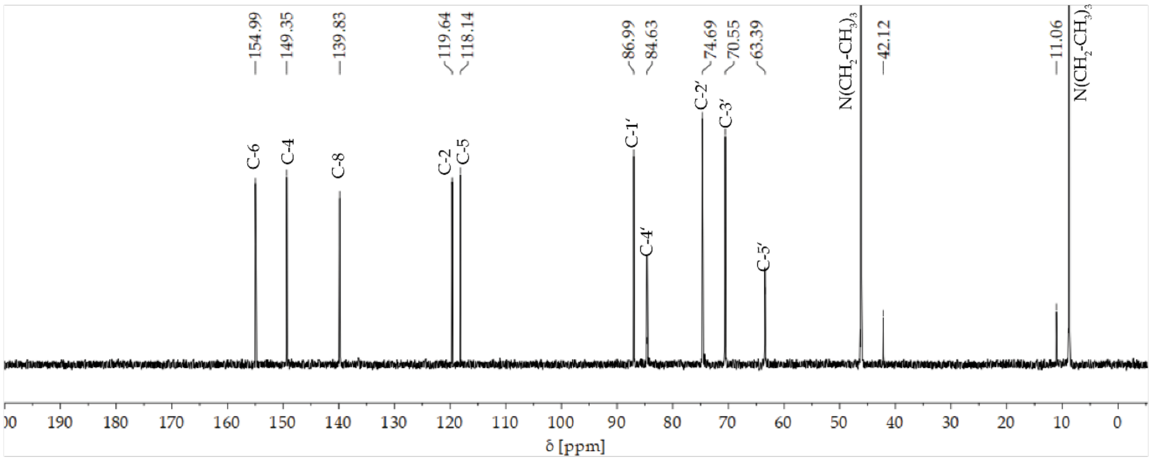


$^{31}P$ NMR (162 MHz, $D_2O$)

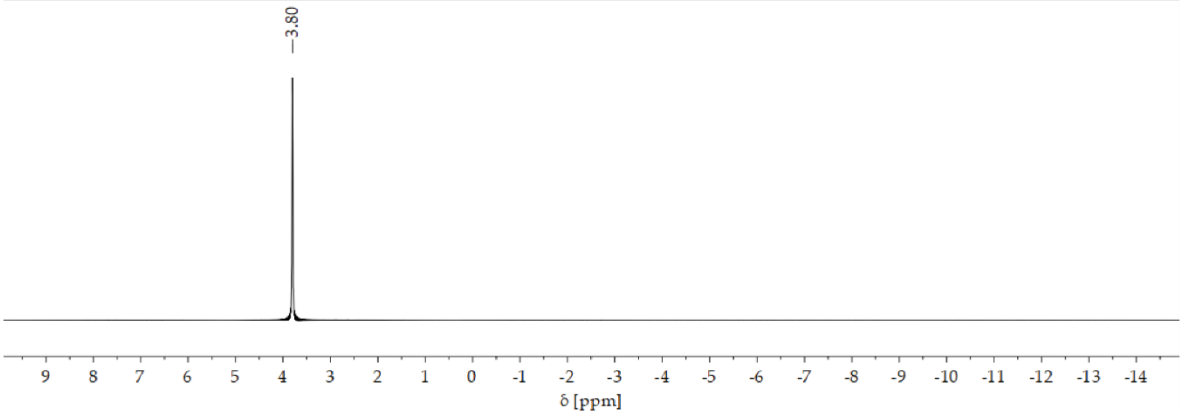

***C*2-(6-aminohexyl)-amino-adenosine 5'-monophosphate (S3)**

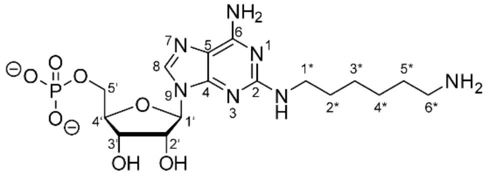

$^{1}H$ NMR (400 MHz, $D_2O$)

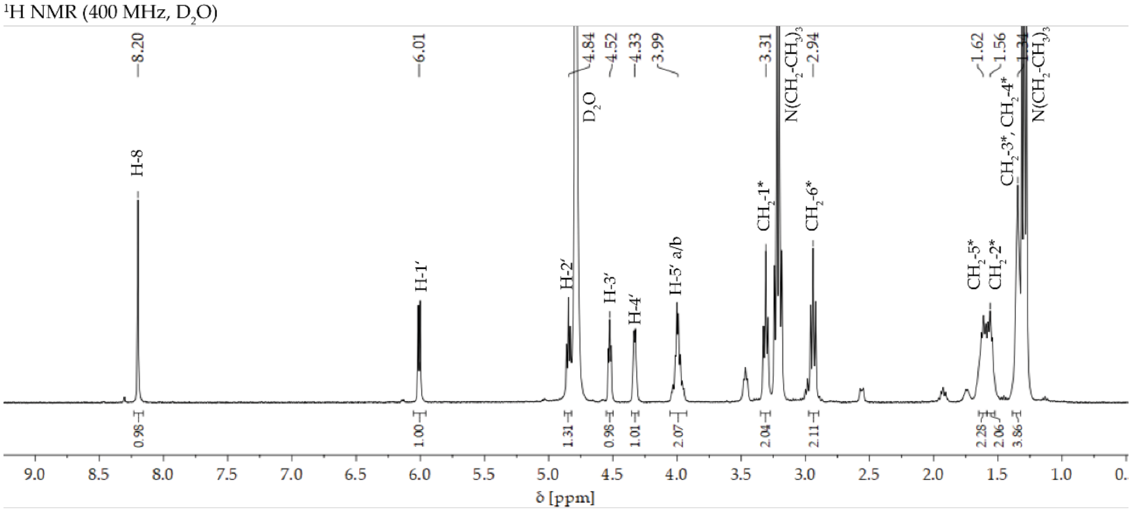


$^{13}C$ NMR (100 MHz, $D_2O$)

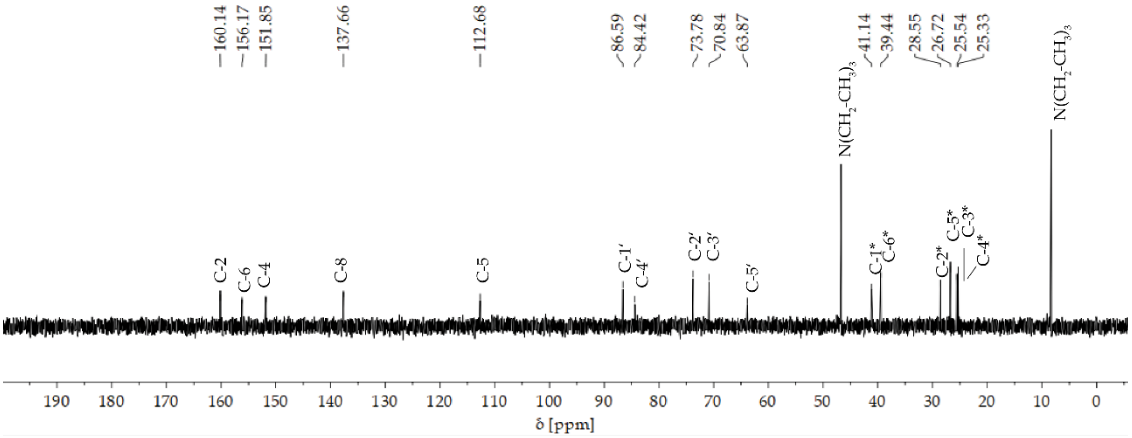


$^{31}P$ NMR (162 MHz, $D_2O$)

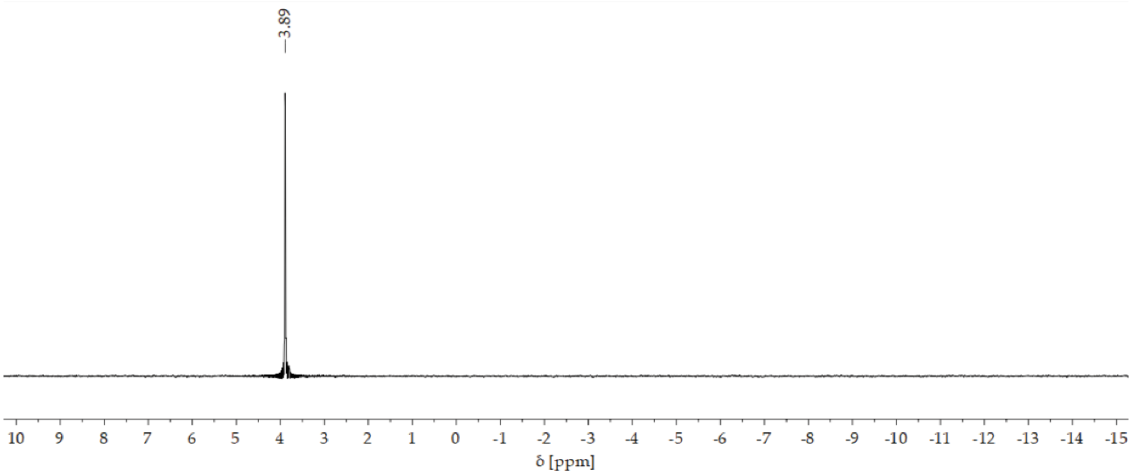

***C*2-(6-(5''-Carboxy-tetramethyl rhodamine)-amidohexyl)-amino adenosine 5'-mono-phosphate (S4, TMR-AMP)**

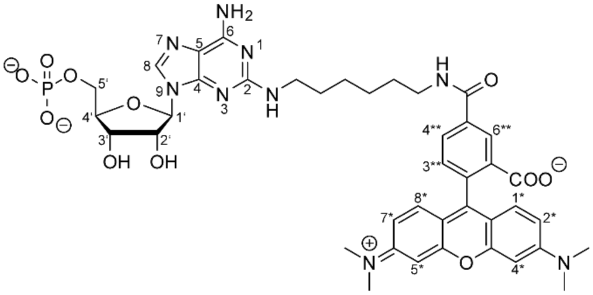


$^{1}H$ NMR (400 MHz, $D_2O$)

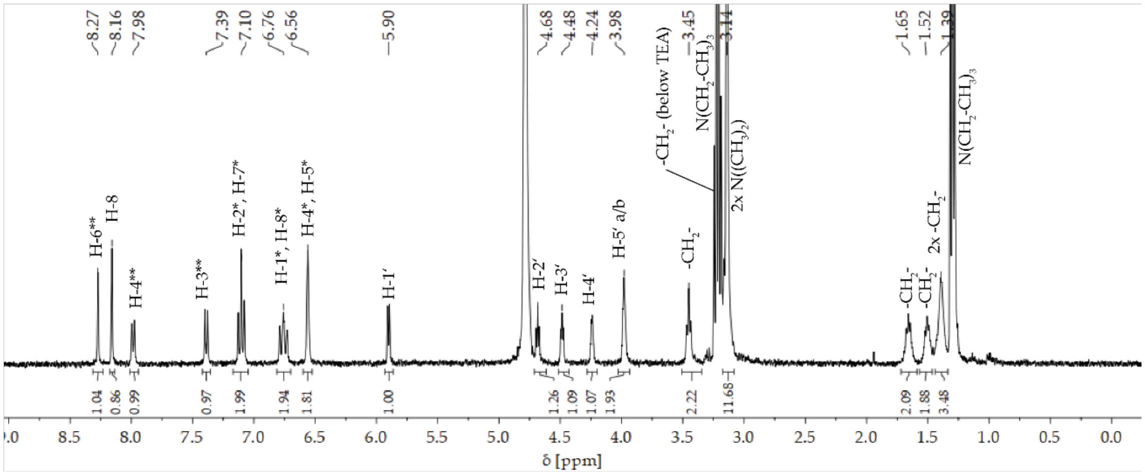


$^{13}C$ NMR (100 MHz, $D_2O$)

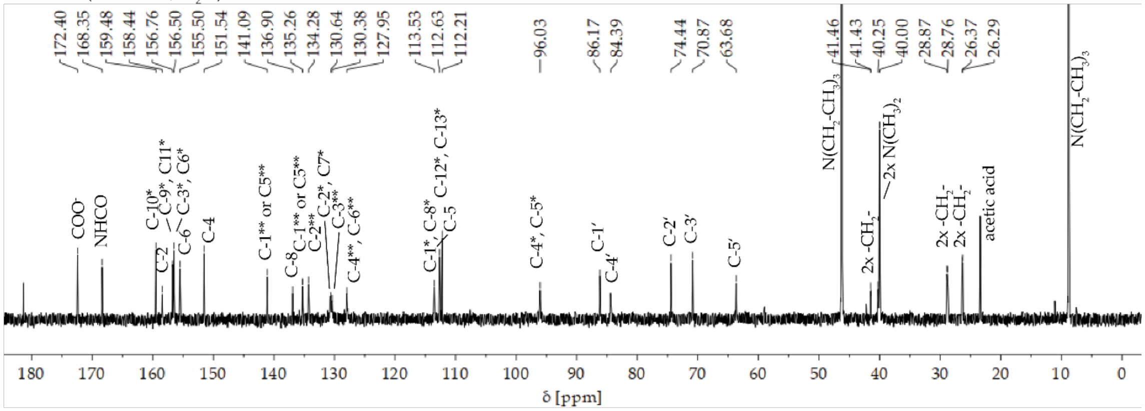


$^{31}P$ NMR (162 MHz, $D_2O$)

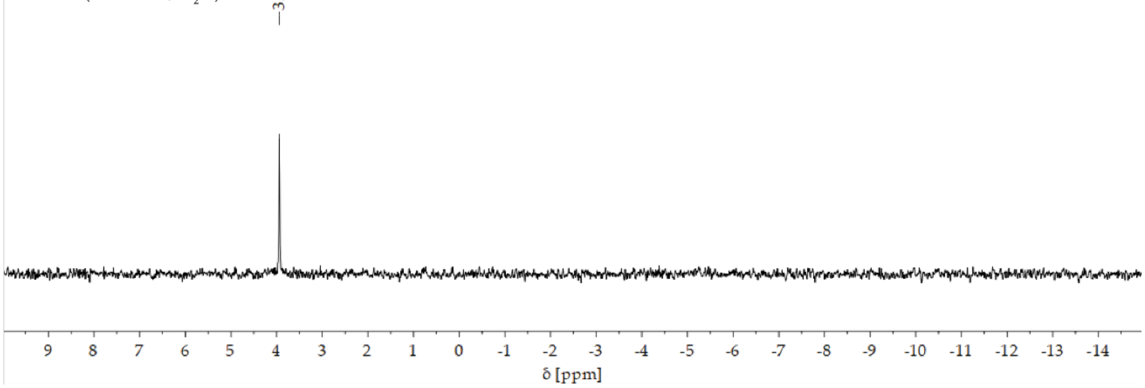

## *C*2-(6-(desthiobiotin)-amidohexyl)-amino adenosine 5'-mono-phosphate (S5, DTB-AMP)

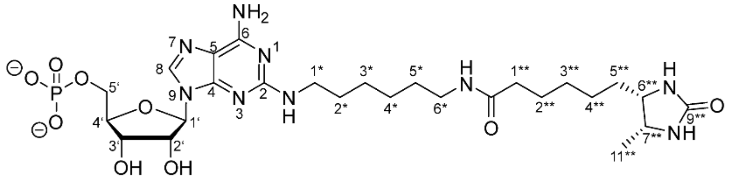


$^{1}$H NMR (400 MHz, $D_2O$)

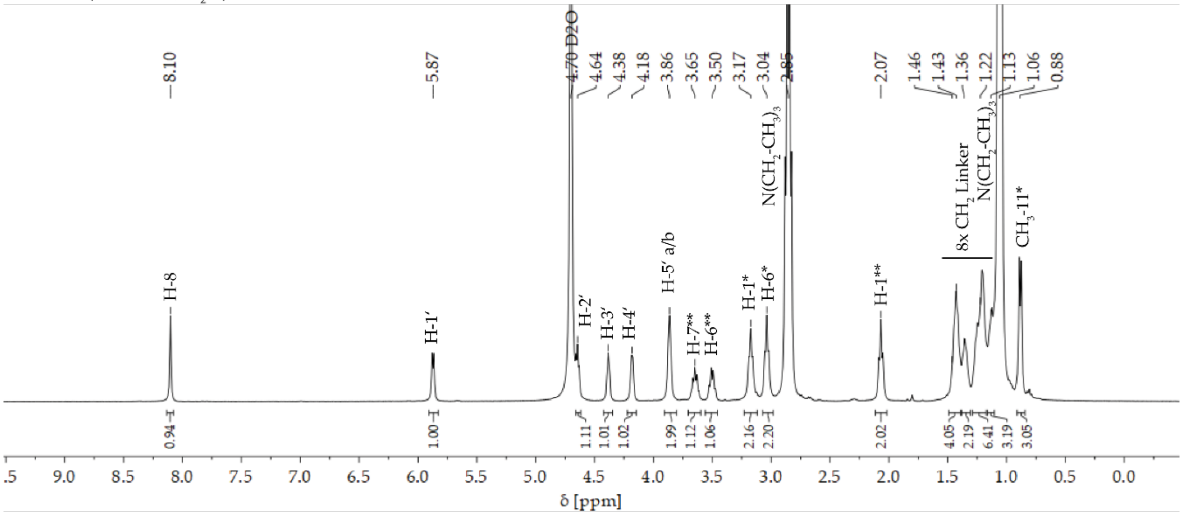


$^{13}$C NMR (100 MHz, $D_2O$)

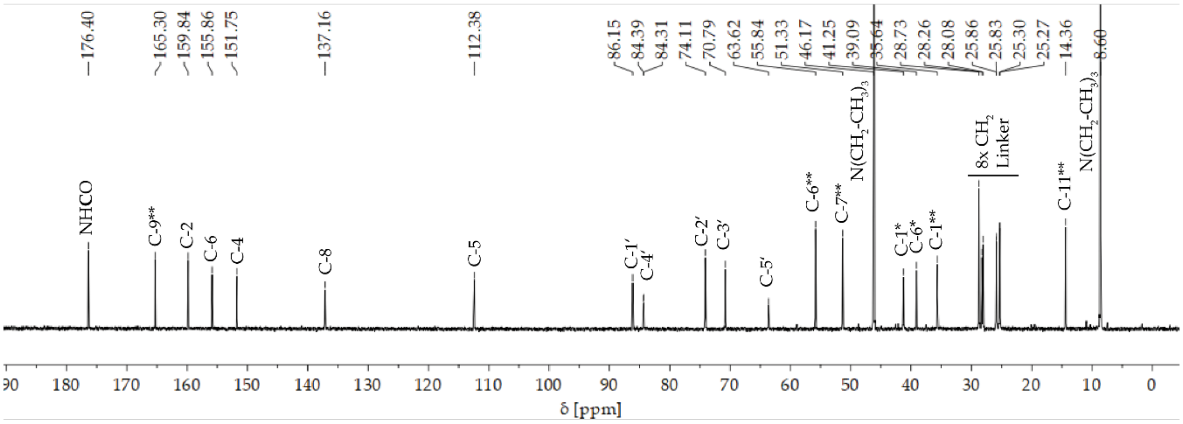


$^{31}$P NMR (162 MHz, $D_2O$)

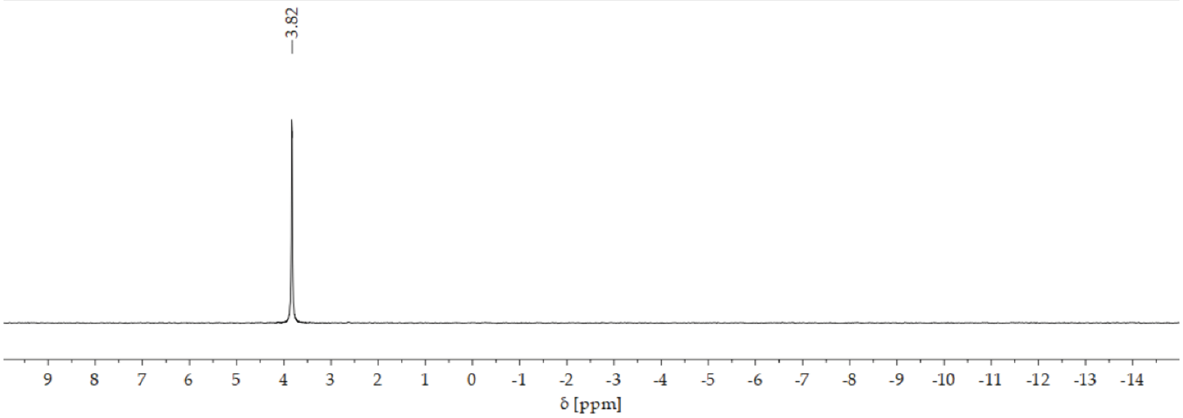

**C2-(6-(5''-Carboxy-tetramethyl rhodamine)-amidohexyl)-amino adenosine nicotinamide dinucleotide (TMR-NAD$^+$ 1)**

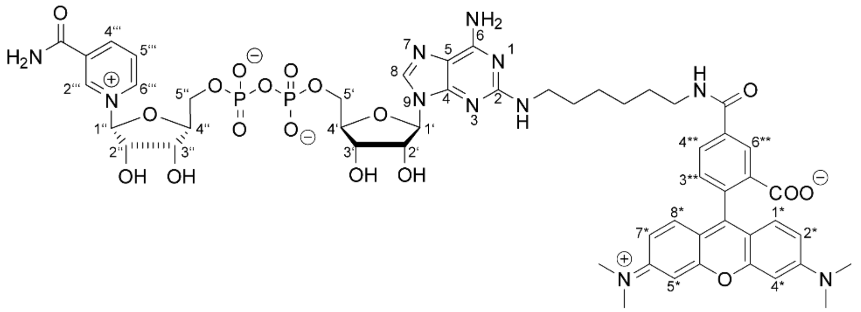

$^{1}$H NMR (400 MHz, $D_2O$)

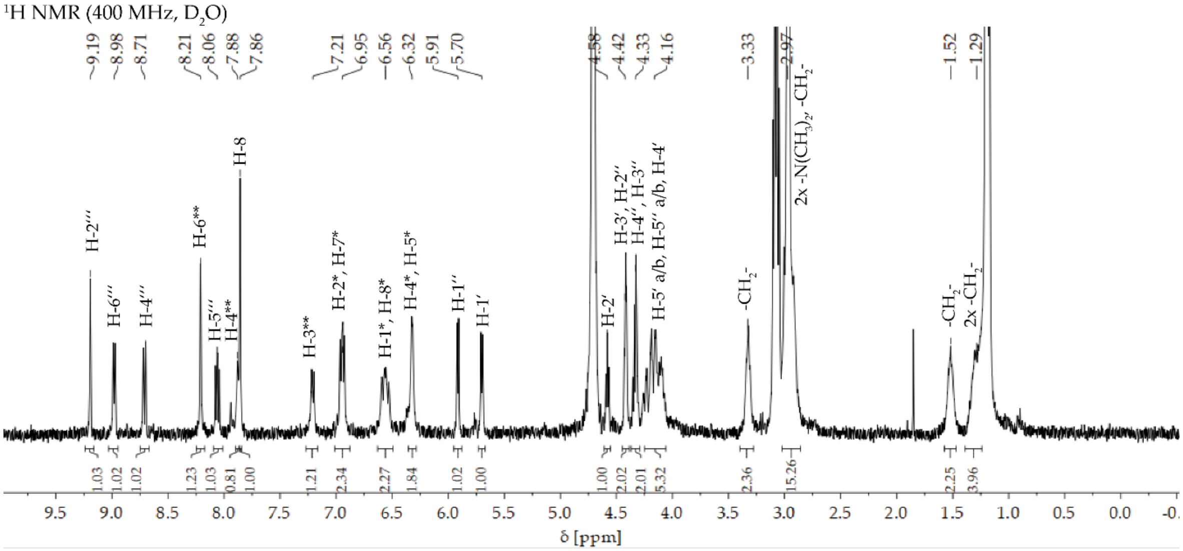


$^{31}$P NMR (162 MHz, $D_2O$)

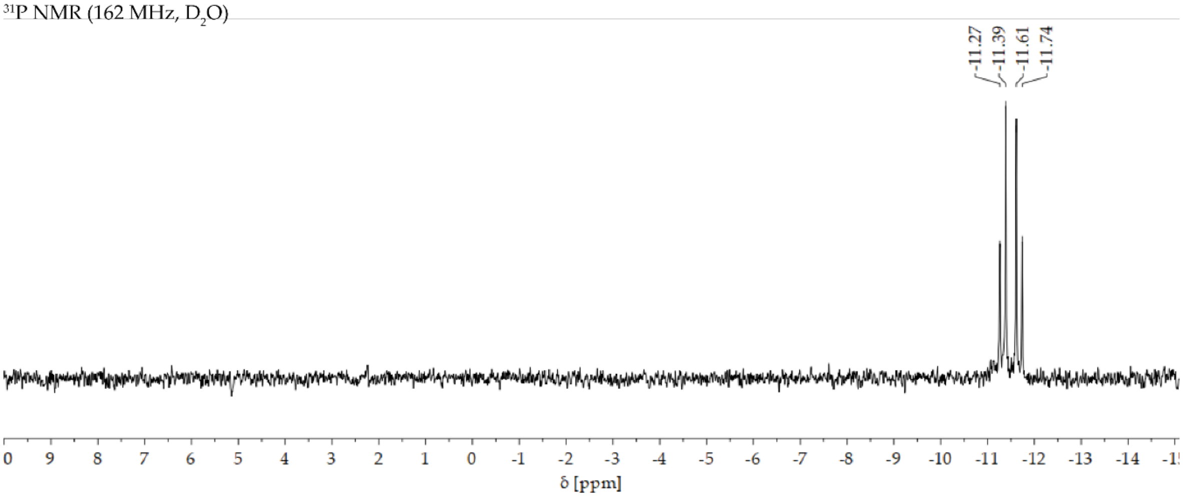

**$C$2-(6-(desthiobiotin)-amidohexyl)-amino adenosine nicotinamide dinucleotide (DTB-NAD$^+$ 2)**

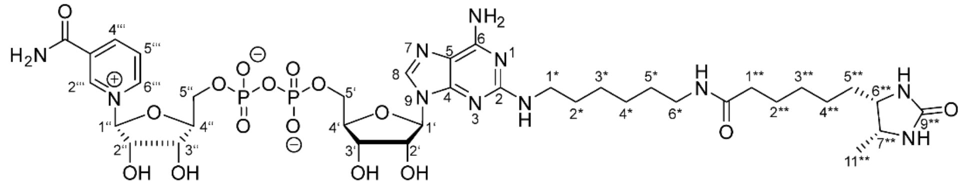

$^1$H NMR (400 MHz, $D_2O$)

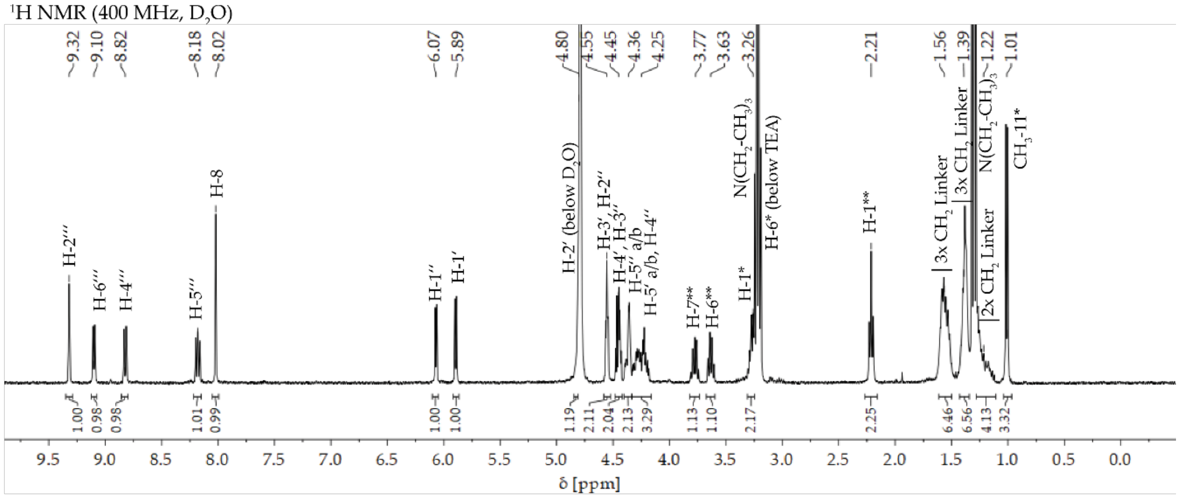


$^{31}$P NMR (162 MHz, $D_2O$)

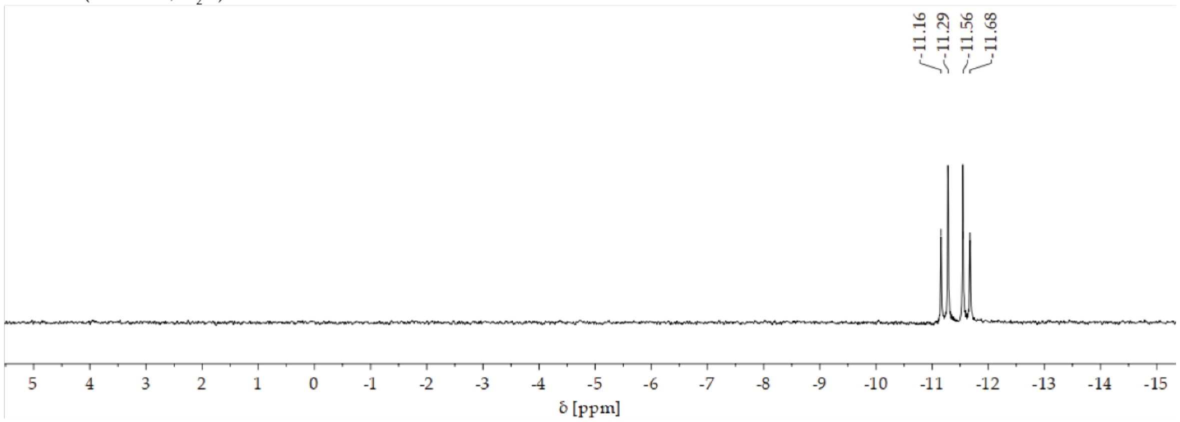

## References


1 Buntz, A. *et al.* Real-Time Cellular Imaging of Protein Poly(ADP-ribos)ylation. *Angew Chem Int Ed Engl* **55**, 11256-11260, doi:10.1002/anie.201605282 (2016).

2 Huang, D. W. *et al.* The DAVID Gene Functional Classification Tool: a novel biological module-centric algorithm to functionally analyze large gene lists. *Genome Biol* **8**, R183, doi:10.1186/gb-2007-8-9-r183 (2007).

3 Gagne, J. P. *et al.* Quantitative proteomics profiling of the poly(ADP-ribose)-related response to genotoxic stress. *Nucleic Acids Res* **40**, 7788-7805, doi:10.1093/nar/gks486 (2012).

4 Martello, R. *et al.* Proteome-wide identification of the endogenous ADP-ribosylome of mammalian cells and tissue. *Nat Commun* **7**, 12917, doi:10.1038/ncomms12917 (2016).

5 Jungmichel, S. *et al.* Proteome-wide identification of poly(ADP-Ribosyl)ation targets in different genotoxic stress responses. *Mol Cell* **52**, 272-285, doi:10.1016/j.molcel.2013.08.026 (2013).

6 Zhang, Y., Wang, J., Ding, M. & Yu, Y. Site-specific characterization of the Asp- and Glu-ADP-ribosylated proteome. *Nat Methods* **10**, 981-984, doi:10.1038/nmeth.2603 (2013).

7 Jiang, H., Kim, J. H., Frizzell, K. M., Kraus, W. L. & Lin, H. Clickable NAD Analogues for Labeling Substrate Proteins of Poly(ADP-ribose) Polymerases. *Journal of the American Chemical Society* **132**, 9363-9372, doi:10.1021/ja101588r (2010).

8 Carter-O'Connell, I., Jin, H., Morgan, R. K., David, L. L. & Cohen, M. S. Engineering the Substrate Specificity of ADP-Ribosyltransferases for Identifying Direct Protein Targets. *Journal of the American Chemical Society* **136**, 5201-5204, doi:10.1021/ja412897a (2014).

9 Gibson, B. A. *et al.* Chemical genetic discovery of PARP targets reveals a role for PARP-1 in transcription elongation. *Science* **353**, 45-50, doi:10.1126/science.aaf7865 (2016).

10 Westcott, N. P., Fernandez, J. P., Molina, H. & Hang, H. C. Chemical proteomics reveals ADP-ribosylation of small GTPases during oxidative stress. *Nat Chem Biol* **13**, 302-308, doi:10.1038/nchembio.2280 (2017).

11 Kalesh, K. *et al.* An Integrated Chemical Proteomics Approach for Quantitative Profiling of Intracellular ADP-Ribosylation. *Sci Rep* **9**, 6655, doi:10.1038/s41598-019-43154-1 (2019).

12 Kvach, M. V. *et al.* Practical synthesis of isomerically pure 5- and 6-carboxytetramethylrhodamines, useful dyes for DNA probes. *Bioconjug Chem* **20**, 1673-1682, doi:10.1021/bc900037b (2009).

13 Menchen, S. M. & Fung, S. 5- and 6- Succinimidyl - carboxylate isomers of rhodamine dyes. DE FR GB SE patent (1992).

14 Hirsch, J. D. *et al.* Easily reversible desthiobiotin binding to streptavidin, avidin, and other biotin-binding proteins: uses for protein labeling, detection, and isolation. *Anal Biochem* **308**, 343-357, doi:10.1016/s0003-2697(02)00201-4 (2002).

15 Wang, Y., Rosner, D., Grzywa, M. & Marx, A. Chain-terminating and clickable NAD(+) analogues for labeling the target proteins of ADP-ribosyltransferases. *Angew Chem Int Ed Engl* **53**, 8159-8162, doi:10.1002/anie.201404431 (2014).

16 Shevchenko, A., Tomas, H., Havlis, J., Olsen, J. V. & Mann, M. In-gel digestion for mass spectrometric characterization of proteins and proteomes. *Nat Protoc* **1**, 2856-2860, doi:10.1038/nprot.2006.468 (2006).

17 Cox, J. *et al.* Accurate Proteome-wide Label-free Quantification by Delayed Normalization and Maximal Peptide Ratio Extraction, Termed MaxLFQ. *Molecular & Cellular Proteomics* **13**, 2513-2526, doi:10.1074/mcp.M113.031591 (2014).

18 Cox, J. & Mann, M. MaxQuant enables high peptide identification rates, individualized p.p.b.-range mass accuracies and proteome-wide protein quantification. *Nat Biotechnol* **26**, 1367-1372, doi:10.1038/nbt.1511 (2008).

19 Tyanova, S. *et al.* The Perseus computational platform for comprehensive analysis of (prote)omics data. *Nature Methods* **13**, 731-740, doi:10.1038/nmeth.3901 (2016).
20 Perez-Riverol, Y. *et al.* The PRIDE database and related tools and resources in 2019: improving support for quantification data. *Nucleic Acids Research* **47**, D442-D450, doi:10.1093/nar/gky1106 (2018).
